# Integrated AMMI, GGE Biplot, and MTSI analyses reveal yield stability and adaptation patterns in cowpea in Ghana

**DOI:** 10.3389/fpls.2026.1897494

**Published:** 2026-10-09

**Authors:** Theophilus Kwabla Tengey, Patrick Attamah, Peter Quandahor, Ophelia Asirifi Amoako, Mariam Seidu, Memunatu Issahaku, Rashid Seidu, Mathew Bapaak Laar, David Nayindow Kpasah, Josiah Darko Osei-Wusu

**Affiliations:** Cowpea Improvement Programme, Council for Scientific and Industrial Research-Savanna Agricultural Research Institute (CSIR-SARI), Nyankpala, Northern Region, Ghana

**Keywords:** genotype × environment interaction, multi-trait selection, Multi-Trait Stability Index, cultivar adaptation, environmental differentiation, drought-prone environments, breeding lines

## Abstract

**Introduction:**

Genotype × environment interaction (G×E) complicates the identification of high-yielding and stable cowpea genotypes across variable production environments. This study evaluated 20 advanced cowpea breeding lines for grain yield, stability, and multi-trait performance across five location-year environments in Ghana during the 2024 and 2025 cropping seasons.

**Methods:**

Trials were conducted using a randomized complete block design with three replications. Both seasons were drought-prone, although moisture stress varied among location-year environments because of erratic rainfall distribution. Grain yield was analysed using additive main effects and multiplicative interaction (AMMI) and genotype plus genotype × environment (GGE) biplot analyses, while grain yield, biomass yield, harvest index, hundred-seed weight, and days to 95% pod maturity were integrated using the Multi-Trait Stability Index (MTSI).

**Results:**

Analysis of variance revealed significant (p < 0.001) effects of environment, genotype, and G×E. The first two interaction principal components explained 92.15% of G×E variation, with Wa_2025 showing the largest IPCA1 score. Tuzievallenga had the highest mean grain yield (1149 kg ha^−1^), whereas IT17K-2030–2 had the lowest AMMI Stability Value (ASV = 114.54). Five genotypes combined high yield with relative stability: Tuzievallenga, IT17K-2030-2, IT17K-1095-2-2, IT17K-849-2-1, and IT17K-1802-1. MTSI values ranged from 2.035 to 5.518, with IT17K-1095-2-2, IT17K-849-2-1, IT17K-1403-1-1, IT17K-2024-4, and Tuzievallenga having the lowest values. IT17K-1095-2-2, IT17K-849-2-1, and Tuzievallenga were common to the top five selections from both approaches. GGE identified one common sector comprising Damongo_2024, Manga_2025, and Nyankpala_2024, with Tuzievallenga as the winning genotype, while Wa_2025 and Nyankpala_2025 formed distinct sectors associated with SARI-tuya and IT17K-1266-2-1, respectively.

**Discussion/Conclusion:**

The findings demonstrate substantial G×E differentiation and identify promising genotypes for farmer-managed on-farm evaluation and complementary use in cowpea breeding.

## 1 Introduction

Cowpea (*Vigna unguiculata* L. Walp) is a major grain legume widely cultivated across sub-Saharan Africa, where it plays a critical role in food security, nutrition, and income generation (Boukar et al., 2019; Feed the Future Innovation Lab for Legume Systems Research, 2024). In Ghana, cowpea is grown across diverse agro-ecological zones, ranging from the Sudan Savanna to the Transition and Guinea Savanna zones (Atakora et al., 2023; Yeboah et al., 2026), and supports the livelihoods of hundreds of thousands of smallholder farmers (Boafo et al., 2016). The high protein content (approximately 25–30%) of cowpea, its contribution to soil fertility through biological nitrogen fixation, and its adaptability to drought-prone environments (Ayalew et al., 2024; Kusi et al., 2025) makes this crop very valuable.

Despite the importance of cowpea, its productivity in Ghana remains relatively low, with average yields ranging from 400 to 600 kg ha^−1^ compared to a potential yield exceeding 2000 kg ha^−1^ under optimal conditions (Horn et al., 2022; Yahaya et al., 2019). Biotic and Abiotic constraints such as drought, poor soil fertility, pests, and diseases have been attributed to this yield gap (Goa et al., 2022). However, a major constraint is the lack of well-adapted varieties that can perform consistently across the country’s highly variable environments (Horn and Shimelis, 2020).

A major challenge in developing improved cowpea varieties is the presence of genotype × environment (G×E) interaction, whereby genotypes perform differently under varying environmental conditions (Belete, 2026). This complicates the identification of widely adapted and high-yielding varieties because the interaction can lead to changes in genotype rankings across locations and seasons (Kusi et al., 2025). When G×E interaction is significant, selection based on a single environment may be misleading. This makes multi-environment trials (METs) essential for reliable evaluation and identification of superior genotypes. It is therefore crucial to understand the nature and magnitude of G×E interaction when developing crop varieties for either broad adaptation or specific adaptation to defined production environments (Saltz et al., 2018). This is particularly relevant to cowpea production in northern Ghana, where rainfall is often erratic and the severity and timing of moisture stress can vary among locations and seasons.

Several statistical approaches have been used in analyzing G×E interaction of which the Additive Main Effects and Multiplicative Interaction (AMMI) model and the genotype plus genotype × environment (GGE) biplot have been the most widely used (Neisse et al., 2018; Rahmati et al., 2024). The AMMI model uses analysis of variance (ANOVA) to partition main effects and uses principal component analysis (PCA) to describe interaction patterns. This provides a robust framework for quantifying stability and identifying genotypes with minimal interaction effects (Rodrigues et al., 2016). Also, the AMMI Stability Value (ASV) enables the ranking of genotypes based on their consistency across environments (Mullualem et al., 2024). GGE biplot analysis focuses on genotype and G×E effects and provides graphical assessment of genotype performance, environmental discrimination and “which-won-where” patterns (Yan et al., 2000, 2007). These two methods together provide complementary insights into genotype performance, stability, and adaptation.

Although AMMI and GGE analyses have been widely applied in cereal crops and some legume crops (Abebe et al., 2024; Atakora et al., 2023; Khan et al., 2021), their combined application to to cowpea breeding materials evaluated across contrasting location-year environments in Ghana remains limited. This necessitates the need to integrate yield stability analysis with environment differentiation and multi-trait selection to provide a more comprehensive assessment of advanced breeding materials. Such an approach is relevant because grain yield alone does not capture all traits contributing to the agronomic and production value of cowpea genotypes.

Cowpea improvement therefore requires simultaneous consideration of grain yield and other agronomically important traits, including maturity, seed size, biomass production and harvest index. Early maturity can contribute to drought escape, seed size is an important agronomic and market trait, biomass has value for livestock feeding, and harvest index provides an indication of the efficiency of assimilate partitioning to grain. The Multi-Trait Stability Index (MTSI) developed by Olivoto et al. (2019) provides a framework for simultaneous selection for mean performance and stability across multiple traits. The method uses factor analysis to summarize trait relationships and calculates the distance of each genotype from a defined ideotype, allowing genotypes to be ranked according to their overall multi-trait performance and stability.

Therefore, this study evaluated 20 advanced cowpea breeding lines across five location-year environments in Ghana with the objectives of: (i) quantifying genotype × environment interaction for grain yield; (ii) identifying high-yielding and stable genotypes using AMMI analysis and the AMMI Stability Value (ASV); (iii) characterizing genotype and environmental responses using GGE biplot analysis; (iv) identifying potential common and environment-specific adaptation patterns and (v) evaluating multi-trait performance and stability using MTSI. The study further sought to identify genotypes combining high yield, stability and desirable agronomic attributes for advancement to farmer-managed on-farm testing and to identify complementary materials with potential value as parents in subsequent cowpea breeding programmes.

## 2 Materials and methods

### 2.1 Plant materials

Twenty cowpea (*Vigna unguiculata* L. Walp) genotypes were evaluated in this study. These materials were selected based on their superior performance from a Regional Coordinated Trial. Fifteen advanced breeding lines were obtained from the CSIR-Savanna Agricultural Research Institute in Ghana, International Institute of Tropical Agriculture (IITA) and INERA-Burkina Faso. Five released varieties were added to the breeding lines for the evaluation. The genotypes represent a range of genetic backgrounds and adaptation patterns relevant to cowpea production in Ghana. Details of genotype, source, and biological status are presented in **Table 1**.

**Table 1:** Cowpea genotypes evaluated in the multi-environment trials, their sources, and biological status.

Genotype	Source	Biological status
IT17K-1016-1-1	IITA	Advanced breeding line
IT17K-1095-2-2	IITA	Advanced breeding line
IT17K-1266-2-1	IITA	Advanced breeding line
IT17K-1279-3-1	IITA	Advanced breeding line
IT17K-1367-2-3	IITA	Advanced breeding line
IT17K-1403-1-1	IITA	Advanced breeding line
IT17K-1707-2-2	IITA	Advanced breeding line
IT17K-1802-1	IITA	Advanced breeding line
IT17K-1809-4	IITA	Advanced breeding line
IT17K-2024-4	IITA	Advanced breeding line
IT17K-2030-2	IITA	Advanced breeding line
IT17K-849-2-1	IITA	Advanced breeding line
IT99K-573-1-1	IITA	Advanced breeding line
KVX782-1	INERA, Burkina Faso	Advanced breeding line
SAC-20KTA-45	CSIR-SARI	Advanced breeding line
Kirkhouse Benga	CSIR-SARI	Released variety
Padi-tuya	CSIR-SARI	Released variety
SARI-tuya	CSIR-SARI	Released variety
Tuzievallenga	CSIR-SARI	Released variety
Wangkae	CSIR-SARI	Released variety

### 2.2 Experimental sites and environments

Field trials were conducted across five environments representing major agro-ecological zones of Ghana during the 2024 and 2025 cropping seasons: Nyankpala_2024, Nyankpala_2025, Damongo_2024, and Wa_2025 (Guinea savanna), and Manga_2025 (Sudan savanna). These environments capture a wide range of production conditions from favourable to stress-prone systems.

The 2024 and 2025 cropping seasons were characterized by below average and erratic rainfall, with more severe drought conditions in 2024, particularly in the Guinea and Sudan savanna zones. These conditions provided an opportunity to evaluate genotype performance under moisture-limited environments typical of smallholder production systems in northern Ghana. Monthly precipitation and temperature for from July to October for 2024 and 2025 cropping seasons compared to a 30-year average from 1994 to 2023 have been presented in **Supplementary Tables S1a, b.**

### 2.3 Experimental design and crop management

The trial was established in each environment using a randomized complete block design (RCBD) with three replications. Each plot consisted of four rows, each 5 m long, with an inter-row spacing of 0.60 m and intra-row spacing of 0.20 m. The net plot area used for yield estimation comprised the two central rows (6 m^2^).

Due to severe moisture limitations during the cropping seasons, particularly in 2024, mineral fertilizer was not applied, as inadequate soil moisture at planting and early growth stages would have limited nutrient uptake efficiency. By the time sufficient rainfall occurred, the optimal window for fertilizer application had passed.

All other agronomic practices were applied uniformly across environments. Weed control was performed manually as required, and insect pests were managed using recommended insecticide applications at flowering and podding stages to minimize yield losses.

### 2.4 Data collection

Data were collected on days to first flowering, days to first pod maturity (D1PM), days to 95% pod maturity (D95PM), pod yield (PY), grain yield (GY), dry biomass yield (BY), harvest index (HI) and one hundred seed weight (HSW). Grain yield was measured at physiological maturity by harvesting plants from the net plot area, followed by threshing, cleaning, and drying. Grain moisture content was adjusted to 12% prior to yield calculation.

Grain yield (GY) was expressed in kg ha^−1^ using the following formula:


$$GY=\frac{\text{Grain weight}\left(\text{kg}\right)}{\text{Net plot area}\left({\text{m}}^{2}\right)}\times 10,000$$

### 2.5 Statistical analysis

All statistical analyses were performed using R software (version 4.3.2; R Core Team, 2023) with relevant packages including *agricolae* for AMMI analysis, *ggplot2*, *dplyr*, *tidyr*, and *ggrepel* for data manipulation and visualization. Significance was assessed at α = 0.05. The GGE biplot analysis was performed using GenStat (version 12; VSN International, 2009).

#### 2.5.1 Analysis of variance

A combined analysis of variance was conducted across the five test environments to evaluate the effects of environment (E), genotype (G), genotype × environment interaction (G×E), and replication nested within environment. The analysis was based on the following linear additive model:


$$Y_{ijk}=\mu +E_{i}+R_{j\left(i\right)}+G_{k}+{\left(GE\right)}_{ik}+\epsilon_{ijk}$$

where: *Y_ijk_* is the observed grain yield of the kth genotype in the jth replication of the ith environment. *μ* is the overall mean. *E_i_* is the effect of the ith environment (i = 1, 2, …, 5). $R_{j\left(i\right)}$ is the effect of replication nested within environment (j = 1, 2, 3). *G_k_* is the effect of the kth genotype (k = 1, 2, …, 20). ${\left(GE\right)}_{ik}$ is the genotype × environment interaction effect. *ϵ_ijk_* is the residual error.

The combined ANOVA included all 20 genotypes and accounted explicitly for replication within environment. The experiment was designed as a 5 × 20 × 3 genotype × environment × replication structure, corresponding to 300 observations when complete. However, inspection of the dataset identified one missing genotype × environment combination, Kirkhouse Benga × Manga_2025, for which observations were unavailable across the three replications. Consequently, the combined analysis was based on 297 observations, resulting in 75 degrees of freedom for G×E, rather than the 76 degrees of freedom expected under a complete 5 × 20 factorial structure. No value was imputed for the missing combination.

For the AMMI analysis, a complete genotype × environment mean matrix was required. Therefore, Kirkhouse Benga, which lacked observations in Manga_2025, was excluded from the AMMI decomposition. The AMMI analysis was consequently conducted using the remaining 19 genotypes evaluated across all five environments, based exclusively on the observed genotype × environment means. This approach avoided introducing imputed values into the estimation of genotype × environment interaction and subsequent stability statistics. The same set of genotypes was also used for GGE Biplot analysis.

#### 2.5.2 AMMI analysis

The Additive Main Effects and Multiplicative Interaction model was used to further partition the G×E interaction into interaction principal component axes (IPCAs). The AMMI model is expressed as:


$$Y_{ij}=\mu +G_{i}+E_{j}+\sum_{k=1}^{n}\lambda_{k}\gamma_{ik}\delta_{jk}+\epsilon_{ij}$$

where *λ_k_* is the singular value of the *k*-th IPCA, and *γ_ik_* and *δ_jk_* are the genotype and environment eigenvectors, respectively.

The G×E interaction matrix was double-centered and subjected to singular value decomposition (SVD) to extract Interaction Principal Component Axes (IPCA). The significance and contribution of each IPCA were evaluated.

#### 2.5.3 AMMI stability value

Genotype stability was quantified using the AMMI Stability Value (ASV), which incorporates contributions from the first two IPCAs:


$$ASV=\sqrt{{\left(\frac{SS_{IPCA1}}{SS_{IPCA2}}\times IPCA1\right)}^{2}+{\left(IPCA2\right)}^{2}}$$

where *SS_IPCA_*_1_ and *SS_IPCA_*_2_ are the sums of squares for the first and second IPCAs, respectively. Lower ASV values indicate greater stability across environments.

#### 2.5.4 Genotype classification

Genotypes were classified based on mean grain yield (above or below the grand mean of 735.8 kg ha^−1^) and AMMI Stability Value (ASV; below median = highly stable, between median and third quartile = moderately stable, above third quartile = less stable). This resulted in six yield–stability groups, ranging from High yield/Highly stable (most desirable) to Low yield/Less stable (least desirable). ASV was calculated following Purchase et al. (2000).

#### 2.5.5 GGE biplot analysis

Genotype plus genotype × environment (GGE) biplot analysis was used to examine genotype performance and genotype × environment (G×E) interaction patterns across the test environments. The genotype and G×E effects were subjected to singular value decomposition, and the first two principal components were used to construct the genotype-focused “which-won-where” biplot. The final GGE biplot was generated using Genstat software. The polygon view of the biplot was used to identify winning genotypes within different environmental sectors and to examine patterns of environmental grouping. Environments occurring within the same sector and sharing a common winning genotype were considered to exhibit similar genotype response patterns, whereas environments occupying separate sectors were interpreted as environment-specific response patterns. These sectors were subsequently assessed as potential mega-environment patterns for genotype adaptation and targeted evaluation.

#### 2.5.6 Multi-trait stability index

The Multi-Trait Stability Index (MTSI) was calculated following Olivoto et al. (2019) using the metan R package (Olivoto and Lúcio, 2020). Genotypic performance and stability across environments were estimated using a linear mixed-model framework, with the Weighted Average of Absolute Scores (WAASB) used to quantify stability. Factor analysis with Varimax rotation was then used to account for correlations among the five complementary traits: grain yield (GY), dry biomass yield (BY), harvest index (HI), hundred-seed weight (HSW), and days to 95% pod maturity (D95PM).

The MTSI was calculated as the Euclidean distance between each genotype and the predefined ideotype in factorial space.


$$MTSI_{i}={\left[\sum_{j=1}^{r}{\left(F_{ij}-F_{j}\right)}^{2}\right]}^{0.5}$$

where *F_ij_* is the score of the ith genotype on the jth factor and *F_j_* is the score of the ideotype.

The ideotype specified maximum values for GY, BY, HI, and HSW and minimum values for D95PM, with lower MTSI values indicating greater proximity to the desired combination of performance and stability (Olivoto et al., 2019). A selection intensity of 20% was used to identify the four genotypes with the most desirable overall multi-trait performance and stability.

## 3 Results

### 3.1 Environmental conditions during the cropping seasons

Precipitation and temperature conditions during the 2024 and 2025 cropping seasons differed from the 1994–2023 long-term averages across the four locations (**Supplementary Tables S1a, b**). During 2024, the combined July–October mean monthly precipitation for Nyankpala and Damongo was 148.90 mm, compared with a long-term mean of 157.89 mm, representing a 5.69% deficit. In 2025, the corresponding mean monthly precipitation across Nyankpala, Manga and Wa was 151.93 mm, compared with 167.61 mm over the long-term period, representing a 9.36% deficit. At individual locations, total July–October precipitation was below the long-term average in both years, with deficits ranging from 3.3% at Damongo in 2024 to 13.9% at Damongo in 2025 (**Supplementary Table S1a**). Nyankpala recorded deficits of 7.8% and 12.2% in 2024 and 2025, respectively, while Manga recorded deficits of 6.0% and 6.7%, and Wa recorded deficits of 7.2% and 9.3%.

The monthly distribution of precipitation also varied among location-years. For example, Nyankpala received 250.54 mm in September 2025, 20.6% above the long-term September average, compared with 189.34 mm in September 2024, which was 8.9% below average. Damongo similarly received 241.86 mm in September 2025 (+30.3%), following substantial deficits in July (−37.7%) and August (−54.3%). At Wa, precipitation was above average in July and September 2025 (+12.9% and +15.8%, respectively), but below average in August and October (−39.8% and −36.3%, respectively). Manga recorded a 20.4% precipitation deficit in October 2025, the largest monthly deficit at the location during that season.

Mean temperatures were generally close too, but slightly above, the long-term averages, with greater departures in 2024 than in 2025 at most locations (**Supplementary Table S1b**). At Nyankpala, mean temperature was 26.59 °C in 2024 and 26.37 °C in 2025, compared with the long-term mean of 26.11 °C, corresponding to increases of 0.48 °C and 0.26 °C, respectively. At Damongo, mean temperature was 26.51 °C and 26.43 °C in 2024 and 2025, respectively, compared with a long-term mean of 25.91 °C, representing increases of 0.60 °C and 0.52 °C. Manga showed the smallest temperature departures, with mean temperatures of 26.77 °C and 26.71 °C compared with a long-term mean of 26.69 °C (+0.07 °C and +0.02 °C). At Wa, mean temperatures were 25.79 °C and 25.46 °C, compared with a long-term mean of 25.42 °C, corresponding to departures of +0.37 °C and +0.04 °C.

Overall, all four locations received below-average July–October precipitation in both cropping seasons, whereas temperature departures were comparatively small and predominantly positive. The magnitude and monthly distribution of precipitation departures varied considerably among location-years. These climatic differences coincided with substantial variation in mean grain yield among the test environments, which ranged from 186.25 kg ha^−1^ in Manga_2025 to 1, 357.84 kg ha^−1^ in Nyankpala_2025.

These contrasting climatic conditions provided the environmental context for interpreting genotype performance, which is presented in the following sections.

### 3.2 Analysis of variance

Grain yield was significantly (p< 0.001) influenced by the environment (E), genotype (G), and genotype × environment interaction (G×E) (**Table 2**). The environment effect, G×E interaction, genotype, and Block(Environment) accounted for 46.62%, 18.27%, 12.51%, and 3.93% of the total sum of squares, respectively, with the residual contributing 18.67%.

**Table 2:** Combined analysis of variance for grain yield across five environments.

Source	df	Sum of squares	Mean square	F-value	P-value	Variance explained (%)	Significance
Environment	4	43, 319, 172.5	10, 829, 793.1	117.40	<0.001	46.62	***
Genotype	19	11, 620, 527.1	611, 606.7	6.63	<0.001	12.51	***
Block (Environment)	10	3, 652, 772.0	365, 277.2	3.96	<0.001	3.93	***
Genotype × Environment	75	16, 975, 495.3	226, 339.9	2.45	<0.001	18.27	***
Error	188	17, 343, 132.9	92, 250.7	—	—	18.67	—
**Total**	**296**	**92, 911, 099.8**	—	—	—	**100.00**	—

df = degrees of freedom. Variance explained (%) was calculated as the sum of squares for each source divided by the total sum of squares × 100. Block effects were nested within environment. ***P< 0.001.

The total dfs, sum of squares and percentage of explained variance have been bolded.

Grain yield across environments was relatively low compared to the crop’s potential, reflecting the drought conditions experienced during the 2024 and 2025 seasons. This indicates the strong influence of environmental stress on genotype performance.

### 3.3 AMMI analysis, interaction partitioning and biplots

The AMMI analysis revealed significant G×E interaction for grain yield. The first two interaction principal component axes (IPCAs) accounted for 68.51% (IPCA1) and 23.64% (IPCA2) of the G×E interaction sum of squares, respectively, explaining 92.15% cumulatively. The overall mean grain yield across the 19 genotypes and five environments was 770.11 kg ha^−1^. The AMMI1 and AMMI2 biplots revealed distinct patterns of genotype and environmental interaction and are presented in Sections 3.3.1 and 3.3.2.

#### 3.3.1 AMMI1 biplot: mean performance and stability

The AMMI1 biplot showed substantial variation in genotype mean grain yield and IPCA1 scores (**Figure 1a**). IT17K-2030-2, IT17K-1095-2-2, and IT17K-1403-1–1 had IPCA1 scores closest to zero, while SARI-tuya, KVX782-1, IT99K-573-1-1, and IT17K-1367-2–3 had positive scores. IT17K-1707-2-2, IT17K-1266-2-1, Wangkae, and IT17K-1016-1–1 had negative scores. Tuzievallenga combined a high mean grain yield of 1149.1 kg ha^−1^ with a relatively small IPCA1 score of 107.31.

**Figure 1:**
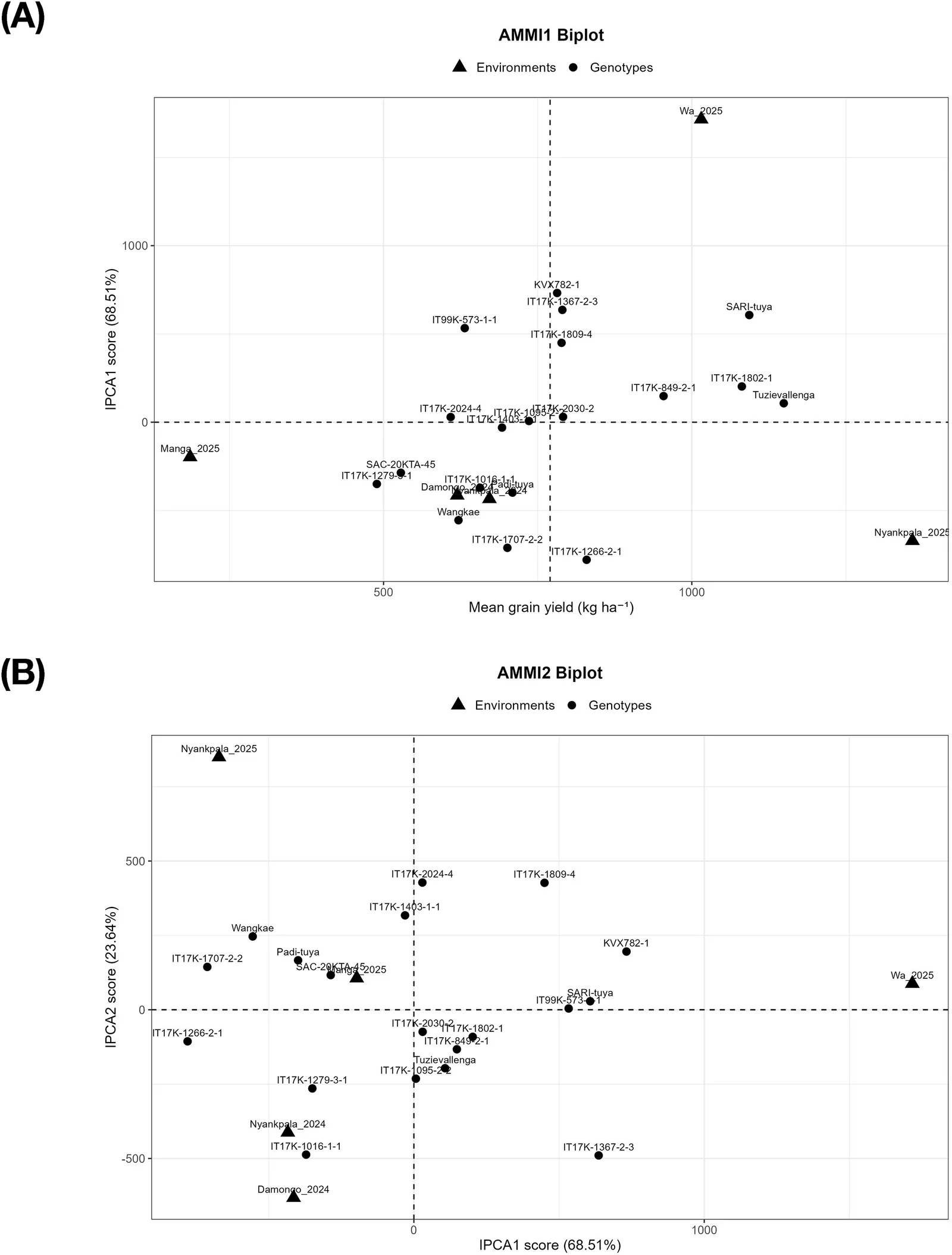
**(a)** AMMI1 biplot for grain yield. AMMI1 biplot showing mean grain yield (kg ha^−1^) against IPCA1 scores for 19 cowpea genotypes evaluated across five environments. IPCA1 explained 68.51% of the G×E interaction variation. **(b)** AMMI2 biplot for grain yield (kg ha^−1^). AMMI2 biplot showing IPCA1 versus IPCA2 scores for 19 cowpea genotypes and five environments. IPCA1 and IPCA2 explained 68.51% and 23.64% of the G×E interaction variation, respectively, accounting for 92.15% cumulatively. Environments and genotypes far from the origin contribute more to the G×E interaction.

#### 3.3.2 AMMI2 biplot: IPCA1 versus IPCA2

The AMMI2 biplot showed distinct distributions of genotypes and environments along IPCA1 and IPCA2 (**Figure 1b**). Damongo_2024, Manga_2025, and Nyankpala_2024 had negative IPCA1 scores. Nyankpala_2025 also had a negative IPCA1 score (−671.41), but a strongly positive IPCA2 score (849.65), whereas Wa_2025 had a markedly positive IPCA1 score (1716.56). IT17K-2030–2 and IT17K-1095-2–2 were positioned relatively close to the origin, while genotypes farther from the origin showed larger interaction scores. The contrasting positions of genotypes and environments demonstrated substantial variation in G×E interaction patterns across the test environments.

### 3.4 Environment IPCA scores and characterization

Mean grain yield and IPCA scores varied substantially among the five test environments (**Table 3**). Nyankpala_2025 recorded the highest mean yield (1357.84 kg ha^−1^), representing the most favourable growing conditions, whereas Manga_2025 had the lowest yield (186.25 kg ha^−1^), reflecting severe stress conditions.

**Table 3:** Mean yield and IPCA scores for five test environments.

Environment	Mean yield (Kg/ha)	IPCA1	IPCA2	IPCA3	IPCA4
Damongo_2024	619.53	-414.26	-631.63	-149.41	-98.38
Manga_2025	186.25	-196.84	106.24	565.03	-8.82
Nyankpala_2024	671.74	-434.05	-412.14	-105.77	120.46
Nyankpala_2025	1357.84	-671.41	849.65	-224.17	-13.64
Wa_2025	1015.21	1716.56	87.87	-85.69	0.37

The environmental IPCA scores showed contrasting contributions to G×E interaction. Damongo_2024 (IPCA1 = −414.26; IPCA2 = −631.63), Manga_2025 (−196.84; 106.24), and Nyankpala_2024 (−434.05; −412.14) had negative IPCA1 scores and occupied a broadly similar region of the AMMI interaction space, although Manga_2025 differed in its IPCA2 score. Nyankpala_2025 also had a negative IPCA1 score (−671.41) but a strongly positive IPCA2 score (849.65), distinguishing it from the other environments. Wa_2025 was markedly differentiated by its strongly positive IPCA1 score (1716.56), the largest absolute IPCA1 value among the environments. These results demonstrate substantial differences among location-year environments in their contribution to G×E interaction.

### 3.5 Genotype IPCA scores and AMMI stability values

Mean grain yield across environments ranged from 489.10 to 1149.15 kg ha^−1^ (**Table 4**). The highest-yielding genotypes were Tuzievallenga (1149.15 kg ha^−1^), SARI-tuya (1093.19 kg ha^−1^), and IT17K-1802-1 (1081.16 kg ha^−1^). Tuzievallenga and SARI-tuya, which were released as cowpea varieties in Ghana in 2024, were therefore among the highest-yielding genotypes in the trial.

**Table 4:** Mean grain yield, IPCA scores, and AMMI Stability Value (ASV) of cowpea genotypes evaluated across five environments in Ghana.

Genotype	Mean yield	IPCA1	IPCA2	IPCA3	IPCA4	ASV
IT17K-2030-2	791.181	30.155	-74.033	13.182	68.484	114.543
IT17K-1095-2-2	735.807	6.835	-231.705	184.288	-14.475	232.550
IT17K-1403-1-1	692.185	-30.443	317.412	-71.319	32.804	329.448
Tuzievallenga	1149.148	107.310	-196.609	-113.726	-4.049	367.959
IT17K-2024-4	608.870	29.208	427.657	177.436	39.207	435.955
IT17K-849-2-1	954.159	148.062	-132.871	-103.690	-12.976	449.242
IT17K-1802-1	1081.159	202.585	-90.999	-432.222	-2.327	594.182
SAC-20KTA-45	528.081	-286.367	116.580	223.415	24.273	838.155
IT17K-1279-3-1	489.100	-349.729	-264.426	126.014	-9.742	1047.578
Padi-tuya	709.148	-398.759	166.384	33.134	-64.617	1167.681
IT17K-1016-1-1	656.293	-371.220	-487.107	27.332	25.423	1181.073
IT17K-1809-4	788.733	449.822	426.752	-110.522	41.474	1371.832
IT99K-573-1-1	631.698	533.054	4.232	99.597	-38.867	1545.014
Wangkae	621.548	-555.128	246.438	-25.946	21.428	1627.751
SARI-tuya	1093.193	607.114	28.521	147.245	-43.978	1759.895
IT17K-1367-2-3	790.037	636.246	-489.760	19.415	33.484	1908.027
IT17K-1707-2-2	700.848	-711.491	144.068	-107.728	-52.208	2067.217
KVX782-1	781.570	732.145	195.653	-16.946	-42.103	2131.055
IT17K-1266-2-1	829.400	-779.399	-106.185	-68.960	-1.236	2261.510

AMMI Stability Value (ASV) varied considerably among genotypes. IT17K-2030–2 had the lowest ASV (114.54), followed by IT17K-1095-2-2 (232.55) and IT17K-1403-1-1 (329.45), indicating relatively small G×E interaction effects. In contrast, KVX782-1 (732.15), IT17K-1266-2-1 (−779.40), and IT17K-1707-2-2 (−711.49) had large absolute IPCA1 scores, indicating stronger interaction with the test environments. The combination of mean yield and ASV showed differences among genotypes in productivity and stability, providing the basis for subsequent classification of genotypes according to yield and stability.

### 3.6 Classification of genotypes by yield and stability

Based on mean grain yield and AMMI Stability Value (ASV), the genotypes were classified into six yield–stability categories (**Supplementary Table S2**). The classification comprised high-yielding/highly stable, high-yielding/moderately stable, high-yielding/less stable, low-yielding/highly stable, low-yielding/moderately stable, and low-yielding/less stable genotypes.

Five genotypes, Tuzievallenga, IT17K-2030-2, IT17K-1095-2-2, IT17K-849-2-1, and IT17K-1802–1 were classified as high-yielding and stable and were therefore identified among the top-performing genotypes based on the combined yield–stability assessment (**Supplementary Table S3**). Tuzievallenga recorded the highest mean grain yield (1149.15 kg ha^−1^) among these genotypes, while IT17K-2030–2 had the lowest ASV (114.54).

Based on grain yield alone, the highest-ranking genotypes were Tuzievallenga, SARI-tuya, IT17K-1802-1, IT17K-849-2-1, IT17K-1266-2-1, IT17K-2030-2, and IT17K-1367-2-3 (**Figure 2**). Thus, the ranking based on grain yield alone differed from the combined yield–stability classification, particularly for genotypes with high yield but relatively large ASV values.

**Figure 2:**
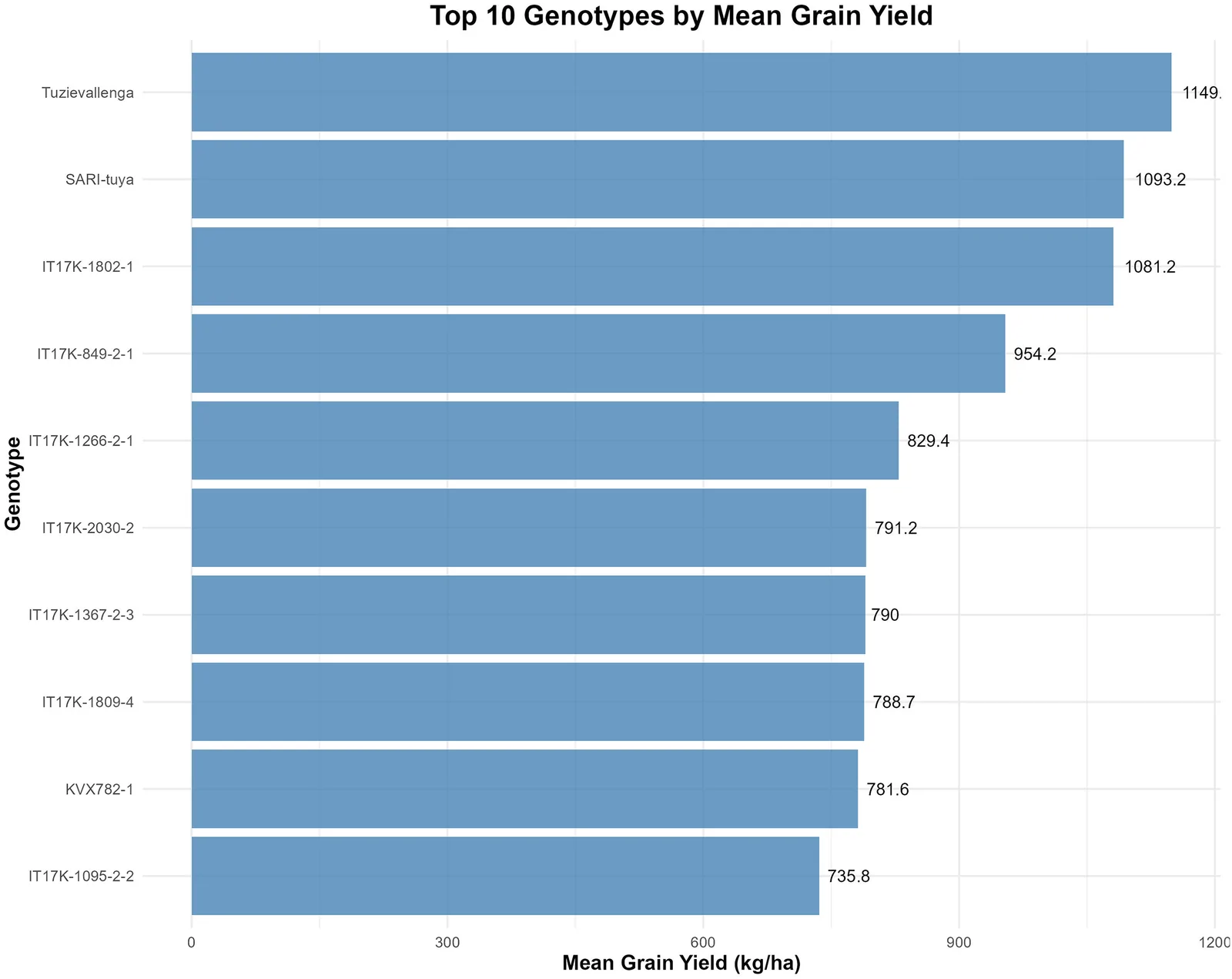
Top 10 cowpea genotypes based on their mean yield.

### 3.7 GGE biplot and potential mega-environment patterns

The genotype-focused “which-won-where” GGE biplot revealed three sectors containing the five test environments (**Figure 3**). Damongo_2024, Manga_2025, and Nyankpala_2024 occurred within the same sector, with Tuzievallenga as the corresponding vertex and winning genotype. Wa_2025 occurred in a separate sector associated with SARI-tuya, whereas Nyankpala_2025 formed another environment-specific sector associated with IT17K-1266-2-1.

**Figure 3:**
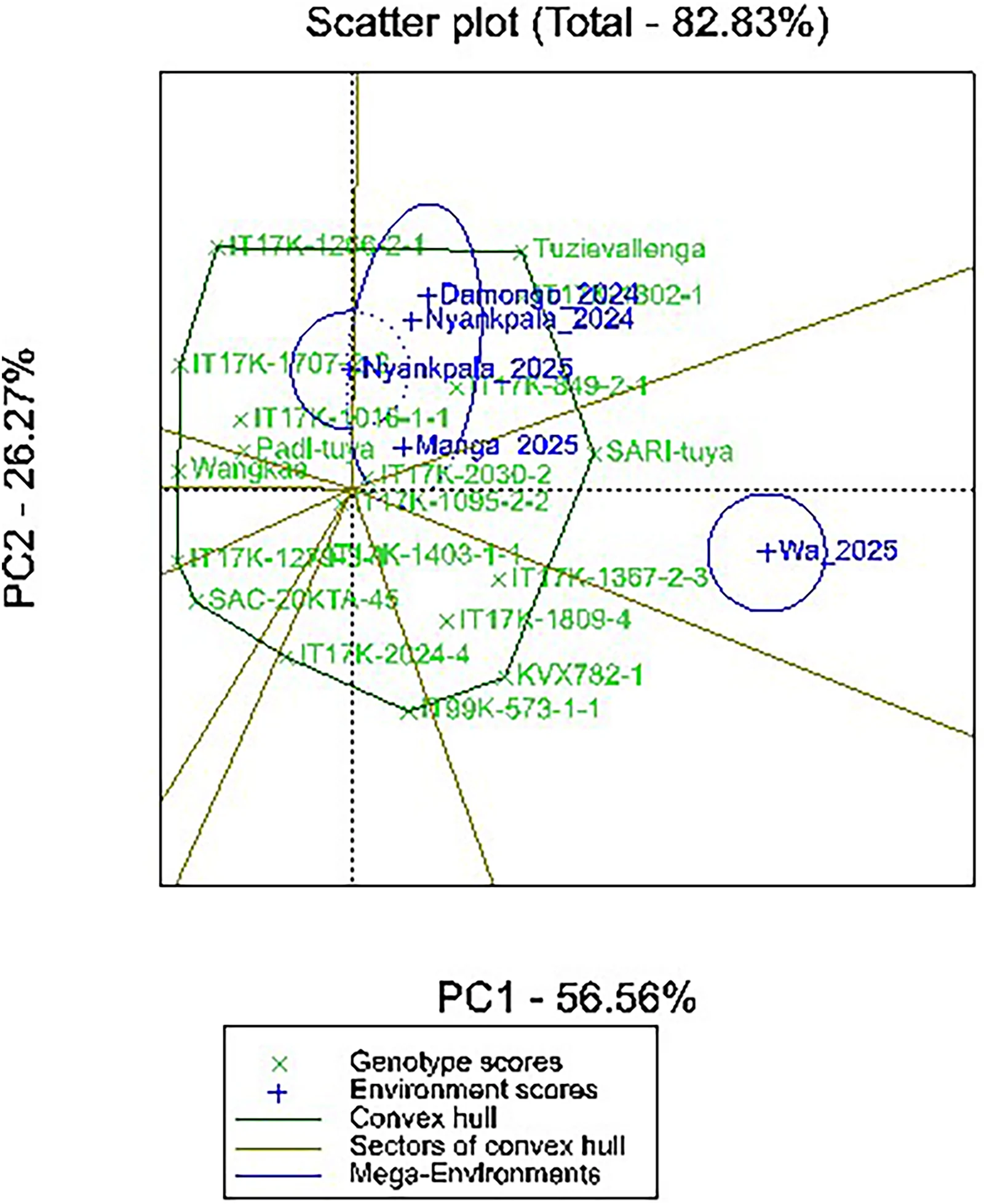
“Which-won-where” GGE biplot of 19 cowpea genotypes across five environments.

The mean grain yields of the environments were 186.25 kg ha^−1^ for Manga_2025, 619.53 kg ha^−1^ for Damongo_2024, 671.74 kg ha^−1^ for Nyankpala_2024, 1015.21 kg ha^−1^ for Wa_2025, and 1357.84 kg ha^−1^ for Nyankpala_2025. The common sector containing Damongo_2024, Manga_2025, and Nyankpala_2024 represented the largest grouping of test environments, while Wa_2025 and Nyankpala_2025 each occurred in sectors containing a single environment.

Overall, the GGE biplot delineated one common sector comprising three environments and two environment-specific sectors, with Tuzievallenga, SARI-tuya, and IT17K-1266-2–1 identified as the respective winning genotypes.

### 3.8 Multi-trait stability index ranking

The Multi-Trait Stability Index (MTSI), calculated using the multi-trait framework of Olivoto et al. (2019) revealed substantial variation among genotypes in their combined performance and stability for grain yield (GY), biomass yield (BY), harvest index (HI), hundred-seed weight (HSW), and days to 95% pod maturity (D95PM) (**Table 5**). MTSI values ranged from 2.035 to 5.518, with lower values indicating greater proximity to the defined breeding ideotype.

**Table 5:** Multi-trait stability index (MTSI) ranking of 20 cowpea genotypes based on grain yield (GY), dry biomass (BY), harvest index (HI), hundred seed weight (HSW), and days to 95% pod maturity (D95PM).

Rank	Genotype	Day to 95% pod maturity	Grain yield (Kg/ha)	Dry biomass yield (Kg/ha)	Harvest index	100 seed weight	MTSI
1	IT17K-1095-2-2	70.7	735.8	2938.3	0.172	17.9	2.035
2	IT17K-849-2-1	69.5	954.2	3345.6	0.183	16.4	2.148
3	IT17K-1403-1-1	71.6	692.2	3139.7	0.148	18.4	2.409
4	IT17K-2024-4	71.7	608.9	2548.3	0.152	17.1	2.723
5	Tuzievallenga	68.1	1149.1	2016.6	0.282	17.3	2.844
6	IT17K-1802-1	68.3	1081.2	3145.6	0.199	19.5	3.277
7	Padi-tuya	71.2	709.1	2896.9	0.157	19.1	3.400
8	SARI-tuya	70.9	1093.2	3032.8	0.234	22.5	3.717
9	IT17K-2030-2	69.8	791.2	3382.2	0.163	22.9	4.006
10	SAC-20KTA-45	70.1	528.1	2867.7	0.13	17.2	4.048
11	IT99K-573-1-1	69.4	631.7	2627.4	0.155	18.6	4.134
12	IT17K-1266-2-1	70.5	829.4	2760	0.162	17.6	4.205
13	IT17K-1279-3-1	71	489.1	3672.4	0.095	17.2	4.257
14	Wangkae	71.1	621.5	2974.9	0.134	19	4.327
15	IT17K-1809-4	70.7	788.7	3293.1	0.168	21.3	4.351
16	IT17K-1707-2-2	70.6	791.7	3158.2	0.153	18.7	4.418
17	IT17K-1016-1-1	68.7	656.3	2554.2	0.154	18.4	4.476
18	Kirkhouse Benga	67	460.6	2317.8	0.13	17.1	4.693
19	KVX782-1	68.9	781.6	3531.7	0.151	18.7	5.172
20	IT17K-1367-2-3	69.7	837.6	2795.7	0.193	18.4	5.518

Lower MTSI (Multi-Trait Stability Index) scores indicate genotypes that are closest to the ideal combination of high performance and stability across multiple traits (Olivoto et al., 2019; Sampaio Filho et al., 2023). Genotypes with lower MTSI values exhibit superior mean performance and greater stability across environments, making them the most desirable for breeding programs and variety recommendation.

The five genotypes with the lowest MTSI values were IT17K-1095-2-2 (2.035), IT17K-849-2-1 (2.148), IT17K-1403-1-1 (2.409), IT17K-2024-4 (2.723), and Tuzievallenga (2.844) [**Figure 4**, **Supplementary Tables S4, S5**)]. These genotypes therefore had the greatest overall proximity to the desired combination of trait performance and stability among the genotypes evaluated.

**Figure 4:**
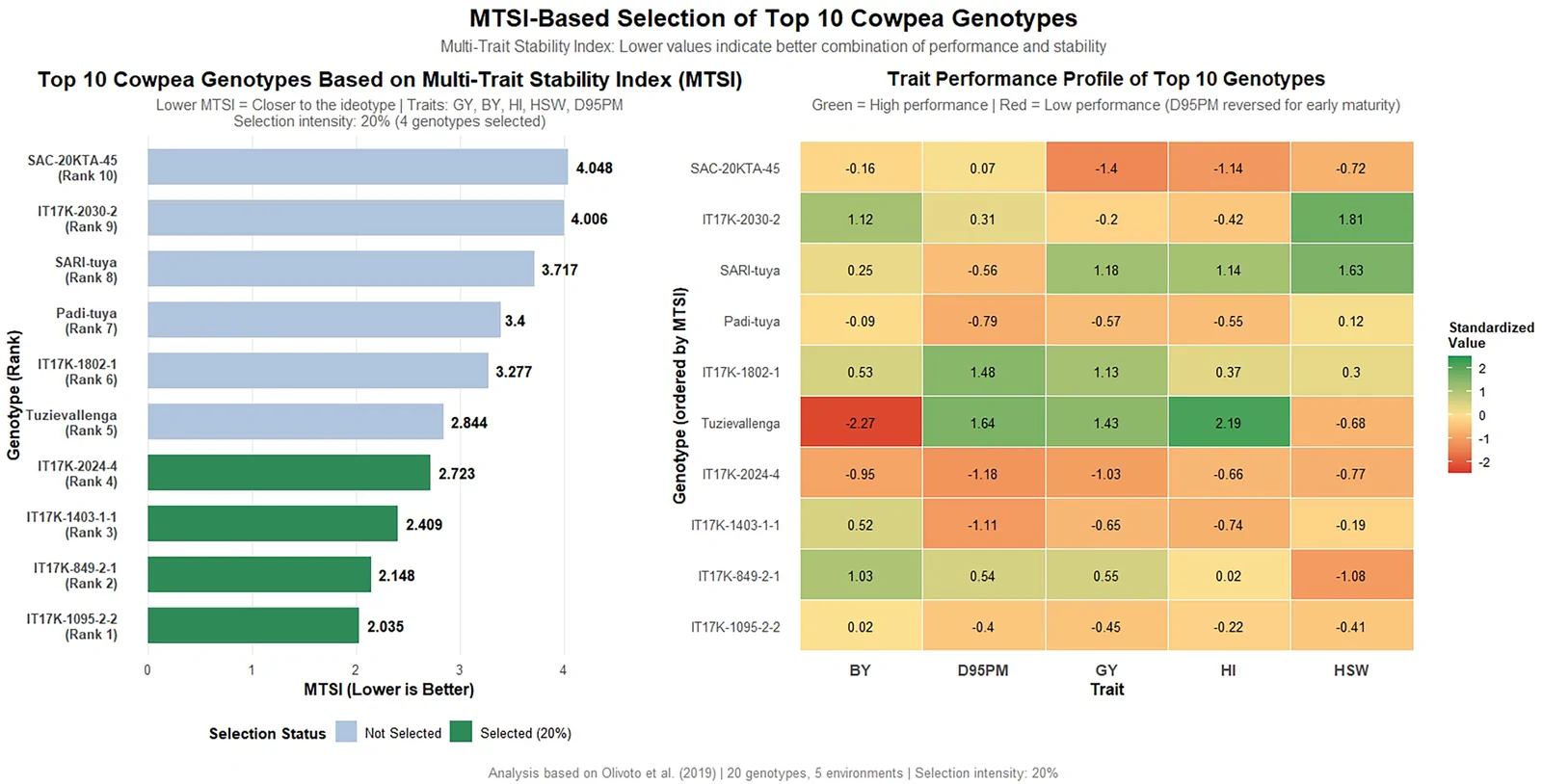
Ideal genotypes based on multi-trait stability index.

The MTSI ranking differed from ranking based on grain yield alone. Tuzievallenga, SARI-tuya, and IT17K-1802-1, which recorded high mean grain yields of 1149.15, 1093.19, and 1081.16 kg ha^−1^, respectively, ranked 5th, 8th, and 6th according to MTSI. Similarly, IT17K-2030-2, which recorded high hundred-seed weight (22.9 g) and biomass yield (3382.2 kg ha^−1^), ranked 9th with an MTSI of 4.006.

In contrast, IT17K-1367-2-3 (5.518), KVX782-1 (5.172), Kirkhouse Benga (4.693), and IT17K-1016-1-1 (4.476) had the highest MTSI values, indicating greater distance from the defined ideotype across the traits considered. Overall, the MTSI ranking differentiated genotypes that performed well across multiple traits from those whose performance was more favourable for individual traits.

### 3.9 Comparison of single-trait and multi-trait selection

Comparison of ASV and MTSI rankings showed substantial but not complete agreement among the 19 genotypes with complete observations (**Supplementary Table S6**). Spearman rank correlation between ASV and MTSI ranks was positive and significant (ρ = 0.763, *P* < 0.001), indicating a strong association between grain-yield stability and multi-trait performance and stability.

Selection based on mean grain yield and AMMI Stability Value (ASV) identified five high-yielding and relatively stable genotypes: Tuzievallenga, IT17K-2030-2, IT17K-1095-2-2, IT17K-849-2-1, and IT17K-1802-1 (**Supplementary Table S3**). In contrast, the five genotypes with the lowest MTSI values were IT17K-1095-2-2, IT17K-849-2-1, IT17K-1403-1-1, IT17K-2024-4, and Tuzievallenga (**Supplementary Table S5**).

Three genotypes IT17K-1095-2-2, IT17K-849-2-1, and Tuzievallenga occurred among the top five genotypes under both selection approaches. IT17K-1403-1–1 and IT17K-2024–4 were among the five lowest-MTSI genotypes but were not included among the five high-yielding and relatively stable genotypes based on mean grain yield and ASV. Conversely, IT17K-2030–2 and IT17K-1802–1 were identified by the yield–ASV criterion but were not among the five genotypes with the lowest MTSI values.

Thus, the two approaches identified three common genotypes and two genotypes unique to each selection criterion. The yield–ASV criterion emphasized grain yield together with yield stability, whereas MTSI integrated grain yield (GY), biomass yield (BY), harvest index (HI), hundred-seed weight (HSW), and days to 95% pod maturity (D95PM) into a single multi-trait selection index.

### 3.10 Summary of key findings

AMMI analysis showed that IPCA1 and IPCA2 accounted for 68.51% and 23.64% of the G×E interaction variance, respectively, explaining 92.15% cumulatively. Among the test environments, Wa_2025 had the largest absolute IPCA1 score (1716.56), while Nyankpala_2025 recorded the highest mean grain yield (1357.84 kg ha^−1^).

Among the genotypes, Tuzievallenga recorded the highest mean grain yield (1149.15 kg ha^−1^), whereas IT17K-2030–2 had the lowest AMMI Stability Value (ASV = 114.54). The GGE which-won-where biplot identified a common sector containing Damongo_2024, Manga_2025, and Nyankpala_2024, with Tuzievallenga as the winning genotype, while Wa_2025 and Nyankpala_2025 occurred in separate environment-specific sectors.

MTSI values ranged from 2.035 to 5.518, with IT17K-1095-2–2 recording the lowest value. Comparison of the yield–ASV and MTSI approaches identified IT17K-1095-2-2, IT17K-849-2-1, and Tuzievallenga among the top five genotypes under both selection criteria.

## 4 Discussion

### 4.1 Magnitude of genotype × environment interaction and implications for cowpea breeding

The combined analysis revealed significant effects of environment, genotype, and genotype × environment interaction (G×E) on grain yield, demonstrating that both the prevailing environmental conditions and genetic differences among the tested materials contributed substantially to variation in performance. Environment accounted for the largest proportion of the total variation in grain yield (46.62%), while G×E accounted for 18.27%. The significant G×E component indicates that genotypic differences were expressed differently across the location-year environments and that the ranking of genotypes was therefore influenced by environmental conditions. This is consistent with previous cowpea studies in which environment and G×E were major sources of variation in grain yield. For example, Goa et al. (2022) reported highly significant environment, genotype and G×E effects, accounting for 42.3%, 23.0% and 34.7% of the total variation, respectively, across moisture-limited environments in southern Ethiopia. In Ghana, Kusi et al. (2025) reported that G×E accounted for 35.12% of the variation in cowpea grain yield across the Deciduous Forest and Sudan Savanna ecologies. Recent work under contrasting water regimes further demonstrated significant genotype, water-regime, and year effects, together with complex interactions for phenological and physiological traits, highlighting the importance of considering both temporal and environmental variation when evaluating drought adaptation in cowpea (Tengey et al., 2026).

The relatively large environmental contribution observed in the present study is expected given the contrasting conditions represented by the five location-year combinations. Mean grain yield ranged from only 186.25 kg ha^−1^ in Manga_2025 to 1357.84 kg ha^−1^ in Nyankpala_2025, indicating substantial differences in the productivity of the testing environments. Both cropping seasons were drought-prone, but the severity of moisture stress differed among location-year combinations because rainfall was erratic in amount and distribution. Thus, the environmental effect should not be interpreted simply as a consequence of differences in total seasonal rainfall. The timing of rainfall relative to crop establishment, flowering and pod filling can be particularly important in determining cowpea productivity under rainfed conditions. Previous multi-environment studies have similarly shown that substantial differences in environmental productivity can lead to pronounced changes in genotype response and consequently affect selection for adaptation and stability (Goa et al., 2022).

The significant G×E interaction has important implications for the interpretation of genotype performance. When G×E is large, the superiority of a genotype in one environment may not necessarily be maintained under another environment, creating difficulties in identifying genotypes with both high productivity and consistent performance. This is particularly relevant for cowpea production systems in northern Ghana, where production environments can differ substantially in rainfall regime and seasonal moisture availability. Recent work in Ghana has likewise demonstrated significant G×E for cowpea grain yield across contrasting ecological environments, with some genotypes showing broad adaptation and others exhibiting advantages in particular environments (Kusi et al., 2025). Similar findings have been reported in other cowpea production regions, where AMMI and GGE analyses have been used to distinguish high-yielding stable genotypes from those exhibiting stronger environmental responses (Gumede et al., 2022).

The AMMI analysis provided further evidence that the G×E interaction was structured and could be meaningfully partitioned into interaction principal component axes. IPCA1 and IPCA2 accounted for 68.51% and 23.64% of the G×E interaction variation, respectively, explaining 92.15% cumulatively. The predominance of the first two axes indicates that most of the systematic interaction pattern could be represented in the AMMI1 and AMMI2 biplots. AMMI is particularly useful in this context because it separates the additive effects of genotype and environment from the multiplicative component of G×E, allowing differences in genotype response to be examined more clearly. The usefulness of AMMI and GGE for interpreting structured G×E and identifying patterns of adaptation in multi-environment trials has been extensively discussed in the crop-breeding literature (Gauch et al., 2008).

The magnitude of G×E observed here therefore indicates that selection based solely on overall mean grain yield would be insufficient for identifying the most reliable breeding materials. A genotype with a high mean yield may achieve that performance because of strong responses in particular environments, whereas another genotype may produce a slightly lower mean but maintain more consistent performance across environments. The objective of stability analysis is consequently not simply to identify genotypes with the smallest interaction, but to determine whether high productivity can be combined with acceptable stability and, where appropriate, useful specific adaptation. This distinction has been emphasized in cowpea studies using AMMI, GGE and stability statistics, where high-yielding genotypes and stable genotypes were not always the same materials (Goa et al., 2022).

Importantly, the presence of significant G×E should not be regarded only as a statistical complication. From a breeding perspective, it can also provide an opportunity to identify genotypes with either broad adaptation or favourable responses to particular production environments. The subsequent AMMI and GGE analyses in this study therefore help to characterize the interaction pattern, while the multi-trait analysis provides an additional basis for selecting genotypes according to a broader breeding objective. This integrated approach is particularly relevant for cowpea improvement because productivity must be considered alongside traits such as maturity, seed size and biomass production.

Overall, the significant environmental, genotypic and G×E effects demonstrate that the tested cowpea materials possessed useful genetic variation but responded differently to the environmental conditions represented in the trial. The relatively large environmental effect confirms the importance of environmental characterization, while the significant G×E demonstrates the need to consider both yield and stability when selecting advanced breeding materials. The following sections therefore examine the environmental patterns and genotype responses in greater detail using AMMI and GGE analyses.

### 4.2 Environmental differentiation and testing strategy

The substantial environmental effect observed in the present study, together with the significant genotype × environment interaction (G×E), demonstrates that the five location-year combinations provided meaningful contrasts for evaluating cowpea performance. Mean grain yield varied markedly from 186.25 kg ha^−1^ in Manga_2025 to 1357.84 kg ha^−1^ in Nyankpala_2025. Such variation indicates that the environmental conditions experienced by the crop differed considerably among location-years and strongly influenced yield expression. Similar environmental effects have been reported in cowpea multi-environment trials. Goa et al. (2022), for example, found that environment accounted for 42.3% of the total variation in grain yield across moisture-limited environments in southern Ethiopia, while Kusi et al. (2025) reported substantial environmental and G×E effects across the Deciduous Forest and Sudan Savanna ecologies of Ghana.

The environmental differences were particularly relevant because both cropping seasons experienced below-average precipitation and above-average temperatures, although rainfall distribution differed between years. The 2024 season received approximately 6.1% less precipitation than the long-term average, whereas the deficit increased to about 10.4% in 2025. Nevertheless, the more favourable temporal distribution of rainfall in 2025 appears to have reduced the severity of moisture limitation in some environments. This distinction is important for interpreting the environmental means because seasonal rainfall totals alone do not necessarily reflect the availability of water during critical crop growth stages. The contrasting performance of the two Nyankpala environments further illustrates the importance of treating location-year combinations as distinct testing environments. Grain yield increased from 671.74 kg ha^−1^ at Nyankpala_2024 to 1357.84 kg ha^−1^ at Nyankpala_2025, despite both seasons being characterized by drought-prone conditions. This difference suggests that drought exposure alone does not adequately describe the growing conditions experienced by the crop. The severity and timing of water stress, particularly in relation to establishment, flowering and pod filling, can substantially influence cowpea productivity. Consequently, differences in rainfall distribution within the season may be as important as differences in seasonal rainfall totals when interpreting environmental effects. Evidence from cowpea production systems in the Sudan Savanna similarly indicates that agricultural drought is influenced not only by precipitation but also by factors such as soil conditions. Recent controlled drought experiments in cowpea have similarly shown that the timing and recurrence of moisture stress can produce complex genotype responses, emphasizing that drought environments should be characterized by both the occurrence and developmental timing of water stress rather than by seasonal moisture availability alone (Tengey et al., 2026).

The AMMI environmental scores provided additional evidence that the location-year environments differed in their contribution to G×E. Wa_2025 had the largest absolute IPCA1 score (1716.56), indicating a particularly strong contribution to the interaction pattern, whereas Nyankpala_2025 combined the highest mean grain yield with a negative IPCA1 score (−671.41) and a strongly positive IPCA2 score (849.65). Damongo_2024, Nyankpala_2024 and Manga_2025 had negative IPCA1 scores, although their IPCA2 scores differed, particularly for Manga_2025. These results demonstrate that environmental differentiation cannot be inferred from yield level alone. An environment may have high productivity while still producing a distinctive pattern of genotype response. Likewise, a large IPCA score indicates a strong contribution to G×E but does not, by itself, establish the specific environmental factor responsible for that interaction. AMMI is therefore most appropriately interpreted together with information on climatic, edaphic and management conditions (Kusi et al., 2025).

The results also support the value of evaluating cowpea genotypes across both locations and years. In Ghana, multi-environment evaluations have demonstrated that genotype performance can differ between agro-ecological zones and between seasons, with some materials showing broad adaptation and others performing particularly well in specific environments. Kusi et al. (2025), for instance, identified both broadly adapted and environment-specific cowpea genotypes when materials were evaluated across the Sudan Savanna and Deciduous Forest zones. Similarly, studies in moisture-limited environments have shown that significant environmental differences can alter genotype rankings and consequently affect the identification of high-yielding and stable materials (Goa et al., 2022).

From a breeding perspective, contrasting environments in the present study were therefore valuable because they exposed the breeding materials to different levels and patterns of environmental constraint. Testing only in a highly favourable environment could favour genotypes with strong yield potential but provide limited information about their consistency under stress. Conversely, testing only under severe stress could favour materials adapted to particular stress conditions without adequately revealing their yield potential under more favourable conditions. A testing network that captures contrasting location-year conditions is more informative for distinguishing broadly adapted genotypes from those with useful specific adaptation. This principle is particularly relevant to cowpea in the Sudan Savanna, where unstable rainfall and other environmental factors can cause substantial variation in productivity among seasons and sites.

The environmental differentiation observed here also has implications for interpretation of the subsequent GGE analysis. The grouping of Damongo_2024, Manga_2025 and Nyankpala_2024 in one GGE sector should not be interpreted as evidence that these environments are identical. Rather, it indicates that they produced a broadly similar genotype discrimination pattern within the current dataset. In contrast, Wa_2025 and Nyankpala_2025 occupied separate sectors, indicating distinctive genotype responses. Because the present evaluation involved only five environments across two cropping seasons, these patterns should be regarded as evidence of potential adaptation patterns rather than definitive delineation of stable mega-environments. Multi-environment testing over additional seasons and farmer-managed conditions would be required before making stronger recommendations about the geographic scope of adaptation.

Overall, the results demonstrate that the environmental component of the trial was not merely a source of experimental variation but an important determinant of genotype expression. The large differences in mean yield, contrasting AMMI environmental scores and year-to-year differences at Nyankpala collectively emphasize the importance of incorporating both spatial and temporal environmental variation into cowpea breeding evaluations. The testing strategy used in this study therefore provided a useful basis for identifying genotypes with broad performance as well as those showing promising responses to particular location-year conditions.

### 4.3 Genotype performance, adaptation and yield stability

The significant genotype effect observed in the present study demonstrates substantial genetic variation for grain yield among the evaluated cowpea materials, providing an important basis for selection. However, the significant G×E interaction indicates that differences among genotypes were not expressed uniformly across environments. This combination of significant genetic variation and differential environmental response is consistent with previous cowpea studies in which genotype, environment and G×E effects significantly influenced grain yield and stability. For example, Goa et al. (2022) reported significant genotype, environment and G×E effects in cowpea evaluated under moisture-limited conditions in southern Ethiopia, while a recent study involving cowpea genotypes across contrasting agro-ecologies of Ghana similarly demonstrated substantial G×E effects and differences in genotype adaptation (Kusi et al., 2025).

Among the genotypes evaluated, Tuzievallenga recorded the highest mean grain yield (1149.15 kg ha^−1^) and had a relatively low ASV (367.96), indicating a favourable combination of productivity and stability. Its position is particularly noteworthy because it was also identified by the GGE analysis as the winning genotype for Damongo_2024, Manga_2025 and Nyankpala_2024. The convergence of high mean yield, relatively small AMMI interaction and GGE superiority across three environments suggests that Tuzievallenga has a comparatively broad pattern of adaptation within the environments represented in this study. Similar studies have shown that genotypes combining high mean performance with relatively low interaction effects are useful candidates for advancement because their performance is less sensitive to environmental variation (Kusi et al., 2025).

SARI-tuya also performed strongly, with a mean grain yield of 1093.19 kg ha^−1^, but its ASV (1759.90) was considerably larger than that of Tuzievallenga. This indicates that its high yield was accompanied by greater interaction with the test environments. The GGE analysis provided further evidence of this response by identifying SARI-tuya as the winning genotype in Wa_2025. Thus, SARI-tuya illustrates an important distinction between high productivity and stability: a genotype can have high yield potential while responding strongly to environmental conditions. Such genotypes may nevertheless be valuable where the target environments resemble those in which they express their greatest advantage. Previous cowpea studies have similarly identified genotypes with strong performance in particular environments alongside genotypes exhibiting broader adaptation, demonstrating that G×E can be exploited both for broad and specific adaptation (Padi, 2004).

In contrast, IT17K-2030–2 had the lowest ASV (114.54), indicating the smallest interaction effect among the genotypes with complete observations. Its position close to the interaction origin is consistent with relatively stable performance across the test environments. However, its mean grain yield was lower than that of the leading genotypes. This contrast highlights the common trade-off between productivity and stability in genotype selection. Stability alone does not necessarily identify the most productive genotype, while selection solely for high yield may favour genotypes with greater environmental sensitivity. The identification of stable but comparatively moderate-yielding genotypes has also been reported in cowpea multi-environment studies, emphasizing the need to consider both mean performance and stability when making breeding decisions (Anyagre et al., 2026).

IT17K-1095-2–2 and IT17K-1403-1–1 also showed relatively low ASV values (232.55 and 329.45, respectively), indicating comparatively small G×E effects. Although these genotypes did not have the highest mean grain yields, their relatively consistent responses across environments make them useful candidates when stability is an important breeding objective. Importantly, their favourable stability rankings were also reflected in the subsequent MTSI analysis, where IT17K-1095-2–2 ranked first and IT17K-1403-1–1 ranked third. The convergence of stability-based and multi-trait selection therefore strengthens their value as breeding materials, although their suitability for direct advancement should also consider the complete trait profile and subsequent on-farm performance.

The contrasting responses of SARI-tuya and IT17K-2030–2 demonstrate why stability should not be interpreted independently of mean performance. SARI-tuya combined high grain yield with relatively large G×E interaction, whereas IT17K-2030–2 showed very low interaction but a lower mean yield. Tuzievallenga occupied a more favourable intermediate position by combining the highest mean yield with relatively low ASV. These differences illustrate that genotype selection depends on the breeding objective: a breeder seeking broad and consistent performance may favour genotypes with small interaction effects, whereas specific adaptation may justify retaining genotypes that respond favourably to particular environments.

The GGE results further indicate that adaptation was not uniform across the testing network. Tuzievallenga was associated with the common sector comprising Damongo_2024, Manga_2025 and Nyankpala_2024, whereas SARI-tuya and IT17K-1266-2–1 were associated with Wa_2025 and Nyankpala_2025, respectively. These patterns should be interpreted as evidence of differential genotype response rather than as definitive proof of environmental specialization. In particular, the sign of an IPCA score alone does not establish adaptation to drought or favourable conditions; adaptation is better inferred from the combination of mean performance, interaction magnitude, GGE relationships and the actual environmental characteristics.

Overall, the results demonstrate that the most desirable genotype depends on whether the breeding objective emphasizes yield, stability, or specific adaptation. Tuzievallenga was particularly notable because of its combination of high yield, relatively favourable stability and repeated GGE superiority. IT17K-2030–2 represented the contrasting stability-oriented phenotype, while SARI-tuya demonstrated high productivity associated with stronger environmental responsiveness. These contrasting profiles provide useful breeding materials for subsequent multi-trait evaluation and for identifying parents with complementary characteristics. Similar evidence from Ghana and other cowpea-growing regions supports the use of AMMI and GGE approaches together to distinguish broadly adapted genotypes from those showing useful environment-specific responses (Kusi et al., 2025). The performance of some genotypes identified in the present study is also supported by recent drought-response evaluations. IT17K-1095-2–2 combined relatively high yield with greater stability under contrasting water regimes, while IT17K-849-2–1 showed a positive yield response under recurrent drought stress, suggesting that these genotypes possess useful attributes for adaptation to water-limited conditions (Tengey et al., 2026).

### 4.4 Yield–stability trade-offs and breeding value of selected genotypes

The combined consideration of grain yield and stability revealed important differences among the genotypes that would not be apparent from mean yield alone. Although **Tuzievallenga** had the highest mean grain yield (1149.15 kg ha^−1^), its ASV of 367.96 placed it fourth among the 19 genotypes with complete observations. This combination of high productivity and relatively low interaction makes Tuzievallenga particularly noteworthy from a breeding perspective. Similar studies in cowpea have shown that genotypes combining high mean yield with low interaction effects are desirable because they are less sensitive to environmental variation and therefore have greater prospects for broad adaptation (Goa et al., 2022; Kusi et al., 2025).

In contrast, IT17K-2030–2 had the lowest ASV (114.54), indicating highly consistent grain-yield performance across the test environments, but its mean grain yield was substantially lower than that of Tuzievallenga and the other leading genotypes. This illustrates a common yield–stability trade-off in genotype selection. A genotype with minimal G×E interaction is not necessarily the highest yielding, while a high-yielding genotype may show greater environmental responsiveness. Consequently, stability statistics should generally be interpreted together with mean performance rather than used as a sole criterion for selection. This principle is supported by cowpea studies in which different stability measures have identified both high-yielding and stable genotypes as well as genotypes with either high yield or high stability but not both simultaneously (Goa et al., 2022; Anyagre et al., 2026).

The performance of SARI-tuya provides another illustration of this trade-off. It recorded the second-highest mean grain yield (1093.19 kg ha^−1^), but its ASV of 1759.90 was considerably higher than those of the most stable genotypes. Its high yield was therefore associated with stronger environmental interaction. The GGE analysis further showed that SARI-tuya was the winning genotype in Wa_2025, suggesting that its high productivity was particularly expressed under the conditions represented by that environment. Such a response should not necessarily be interpreted as a weakness; rather, it indicates that genotypes with stronger interaction may have value where the target environment consistently favours their expression. Previous cowpea studies in Ghana have similarly identified genotypes with specific environmental advantages alongside broadly adapted materials, demonstrating that both broad and specific adaptation can be useful breeding objectives (Kusi et al., 2025).

The relatively favourable combination of yield and stability observed for Tuzievallenga is particularly important because it was also identified as the GGE winner in three environments: Damongo_2024, Manga_2025 and Nyankpala_2024. This convergence between mean performance, ASV and GGE results provides stronger evidence for its consistent performance than any single analytical criterion considered independently. However, interpretation should remain within the scope of the present testing environments. The results demonstrate favourable performance across the environments evaluated, but they do not establish universal adaptation beyond the environmental range represented in the study.

The contrasting profiles of Tuzievallenga, IT17K-2030–2 and SARI-tuya demonstrate that breeding value cannot be defined by yield or stability alone. Tuzievallenga represents a favourable combination of high yield and relatively low interaction; IT17K-2030–2 represents strong stability but more moderate yield; and SARI-tuya represents high yield accompanied by stronger environmental responsiveness. These differences can be exploited according to breeding objectives. A genotype combining high yield and stability may be prioritized for advancement, whereas a stable moderate-yielding genotype may provide useful genetic material for improving consistency, and a highly responsive genotype may be valuable where its favourable environmental association can be reproduced.

This distinction is particularly relevant in the context of cowpea production in Ghana, where production environments vary in rainfall, temperature and other environmental conditions. Recent work in Ghana has demonstrated significant G×E interaction among cowpea genotypes evaluated across contrasting agro-ecological environments and has shown that some genotypes exhibit broad adaptation whereas others perform particularly well in specific environments (Kusi et al., 2025). The present results extend this evidence within the northern Ghana testing environments by showing that yield performance, stability and specific adaptation can lead to different rankings of the same breeding materials.

Overall, the yield–stability analysis suggests that Tuzievallenga is the strongest candidate when high productivity and relatively favourable stability are considered jointly, whereas IT17K-2030–2 provides a contrasting source of strong yield stability. SARI-tuya, despite its higher ASV, remains important because of its high yield and demonstrated superiority in Wa_2025. These contrasting profiles provide useful material for subsequent multi-trait selection and breeding, where complementary attributes can be combined rather than relying on a single stability criterion.

### 4.5 Environmental differentiation and potential mega-environment patterns

The AMMI and GGE analyses provided complementary perspectives on the environmental structure of the genotype × environment interaction. Whereas AMMI partitioned the interaction into principal components and showed substantial differences among the location-year environments, the genotype-focused GGE biplot provided a clearer representation of which genotypes were associated with the highest performance in different environmental sectors. GGE analysis is particularly useful for examining genotype evaluation and potential mega-environment patterns because it combines the genotype main effect with the G×E interaction after removing the environmental main effect (Yan et al., 2000, 2007).

The present GGE analysis identified three distinct sectors containing the five environments. Damongo_2024, Manga_2025 and Nyankpala_2024 occurred within a common sector, with Tuzievallenga as the corresponding winning genotype. In contrast, Wa_2025 and Nyankpala_2025 each occupied separate sectors, associated with SARI-tuya and IT17K-1266-2-1, respectively. The common sector is noteworthy because it indicates a broadly similar pattern of genotype response among three otherwise contrasting location-year environments. However, it should be interpreted as a potential common adaptation pattern rather than a definitive mega-environment, particularly because the analysis involved only five environments and two cropping seasons.

The association of Tuzievallenga with Damongo_2024, Manga_2025 and Nyankpala_2024 is consistent with its strong overall performance and relatively favourable stability. Its identification as the winning genotype across these three environments provides additional evidence that its performance was comparatively consistent within the portion of the testing network represented by this sector. Similar use of GGE polygon analysis has enabled cowpea researchers in Ghana to distinguish environments with common genotype responses and to identify genotypes showing broad or specific adaptation. Kusi et al. (2025), for example, identified three potential mega-environments among four cowpea testing environments in the Deciduous Forest and Sudan Savanna zones of Ghana.

The two environment-specific sectors provide a different perspective on adaptation. SARI-tuya was the winning genotype in Wa_2025, which also had the largest absolute IPCA1 score in the AMMI analysis. This correspondence suggests that Wa_2025 imposed a distinctive pattern of genotype response and that SARI-tuya expressed a relative advantage under those particular conditions. Similarly, IT17K-1266-2–1 was associated with Nyankpala_2025, an environment characterized by the highest mean grain yield but a distinctive AMMI interaction profile, including a strongly positive IPCA2 score. These findings demonstrate that high environmental productivity does not necessarily correspond to a single general pattern of genotype response.

An important feature of the results is the different classification of Nyankpala across years. Nyankpala_2024 occurred in the common sector with Damongo_2024 and Manga_2025, whereas Nyankpala_2025 occupied a separate sector associated with IT17K-1266-2-1. This contrast emphasizes that the environmental unit relevant to genotype adaptation is the location-year combination, rather than location alone. Differences in rainfall distribution, temperature and other seasonal conditions can alter the relative ranking of genotypes at the same geographical location. Consequently, classifying environments solely according to geographical location could obscure important temporal variation in genotype response.

The identification of a common sector and two environment-specific sectors also illustrate the value of combining AMMI and GGE analyses. The two approaches do not constitute independent validation of one another; rather, they provide different representations of the same underlying G×E structure. AMMI was useful for quantifying the interaction and identifying environments and genotypes with large or small interaction effects, whereas GGE provided a direct visualization of genotype performance and potential environment-specific winners. Previous methodological comparisons have emphasized that both AMMI and GGE can be used for mega-environment investigation, although their graphical representations and interpretations differ (Yan et al., 2007; Gauch et al., 2008).

The present results therefore provide preliminary evidence of environmental differentiation within the Northern Ghana testing network. The common sector involving Damongo_2024, Manga_2025 and Nyankpala_2024 may represent a potential adaptation zone for genotypes such as Tuzievallenga, while Wa_2025 and Nyankpala_2025 showed more environment-specific genotype responses. Nevertheless, the limited number of environments and years warrants caution against treating these sectors as fixed mega-environments. Further testing under farmer-managed conditions would be useful for determining whether the observed patterns are sufficiently repeatable to support environment-specific cultivar recommendations.

### 4.6 Multi-trait selection index and farmer-relevant traits

Selection based only on grain yield and its stability may overlook other characteristics that influence the agronomic and practical value of cowpea genotypes. In the present study, the Multi-Trait Stability Index (MTSI) was therefore used to integrate grain yield (GY), biomass yield (BY), harvest index (HI), hundred-seed weight (HSW), and days to 95% maturity (D95PM). The MTSI approach of Olivoto et al. (2019) was developed specifically to facilitate simultaneous selection for mean performance and stability across multiple traits in multi-environment trials, thereby providing an alternative to selecting genotypes independently for each trait.

The MTSI values in the present study ranged from 2.035 to 5.518, with lower values indicating greater proximity to the specified ideotype. IT17K-1095-2-2, IT17K-849-2-1, IT17K-1403-1-1, IT17K-2024-4, and Tuzievallenga had the five lowest MTSI values. The identification of these genotypes indicates that their overall performance was favourable when the five traits were considered simultaneously, rather than on the basis of grain yield alone. This distinction is important because selection for a single trait can result in the advancement of genotypes that perform well for one characteristic but have undesirable performance for other traits. Multi-trait selection indices have increasingly been used to address this limitation by combining several breeding objectives into a single selection criterion (Olivoto et al., 2019).

The relative positions of the released varieties provide an informative illustration of the difference between yield-based and multi-trait selection. Tuzievallenga, which recorded the highest mean grain yield (1149.15 kg ha^−1^), ranked fifth for MTSI, whereas SARI-tuya, with the second-highest mean grain yield (1093.19 kg ha^−1^), ranked eighth. Thus, high grain yield did not necessarily translate into the closest overall match to the multi-trait ideotype. For Tuzievallenga, its fifth position indicates that its high yield was accompanied by a generally favourable combination of the other traits included in the index. In contrast, the lower MTSI position of SARI-tuya suggests that its high grain yield was offset by less favourable performance and/or stability for one or more of the other traits.

The results also demonstrate the importance of considering biomass and seed characteristics alongside grain yield. IT17K-2030-2, for example, had the lowest ASV and therefore exhibited the greatest grain-yield stability, while also recording relatively high biomass yield (3382.2 kg ha^−1^) and hundred-seed weight (22.9 g). Nevertheless, it ranked ninth according to MTSI. This result illustrates that strong performance for individual traits does not necessarily result in a high overall multi-trait ranking. Rather, MTSI evaluates the combined distance of a genotype from the desired trait profile. Similar observations have been reported in recent multi-trait studies, where genotypes with outstanding performance for particular traits were not always the genotypes selected when multiple objectives were considered simultaneously (Abdou et al., 2026).

Seed size and maturity are particularly relevant traits in cowpea improvement because they contribute to cultivar differentiation and can influence the suitability of varieties for different production and market contexts. Hundred-seed weight provides an important measure of seed size, while maturity duration is relevant to production in environments where the growing season is constrained by intermittent rainfall. Biomass yield is also relevant to dual-purpose cowpea systems in which both grain and vegetative material contribute to the value of the crop. Recent cowpea research has similarly emphasized the importance of jointly evaluating grain yield, fodder or biomass production, seed size and maturity when selecting genotypes for variable production environments (Abdou et al., 2026). The importance of integrating maturity and productivity traits is further supported by recent drought-response work in cowpea, where days to 95% pod maturity was negatively associated with grain yield under recurrent drought stress, indicating the importance of early maturity as a drought-escape mechanism (Tengey et al., 2026).

The MTSI results therefore provide a different perspective from the yield–stability analysis. IT17K-1095-2–2 and IT17K-849-2–1 ranked highly under both approaches, while IT17K-1403-1–1 and IT17K-2024–4 were particularly favoured by the multi-trait index. Tuzievallenga also remained among the leading genotypes under both approaches, although it was not the first-ranked genotype under MTSI. This partial convergence suggests that some genotypes combine desirable grain-yield performance and stability with favourable secondary traits, whereas others may be preferred depending on the relative breeding objectives.

Importantly, MTSI should be viewed as a decision-support tool rather than a substitute for breeder judgement. The ranking depends on the traits included, the direction of desirability specified for each trait, and the ideotype against which genotypes are evaluated. Olivoto et al. (2019) emphasized that multi-trait selection is most useful when breeding objectives are explicitly defined and multiple traits are considered simultaneously. In the present study, the index provides evidence that selection based solely on grain yield or grain-yield stability would not necessarily identify the same genotypes as selection based on a broader agronomic objective.

Overall, the MTSI analysis demonstrates the value of integrating yield, biomass production, harvest index, seed size and maturity when evaluating cowpea breeding materials. The results support particular attention to IT17K-1095-2-2, IT17K-849-2-1, IT17K-1403-1-1, IT17K-2024–4 and Tuzievallenga, while recognizing that genotypes with strengths in individual traits may also have value as breeding parents. The comparison between MTSI and AMMI/ASV is considered further in Section 4.7.

### 4.7 Comparison of selection methods: AMMI/ASV and MTSI

The comparison of AMMI-based stability selection and the Multi-Trait Stability Index (MTSI) illustrates how the choice of selection criterion can influence the identification of promising cowpea genotypes. AMMI and ASV primarily describe genotype performance and stability for grain yield, whereas MTSI integrates performance and stability across several traits simultaneously. The MTSI was specifically developed to facilitate simultaneous selection for mean performance and stability across multiple traits in multi-environment trials (Olivoto et al., 2019).

There was considerable agreement between the two approaches. Among the 19 genotypes with complete observations, the Spearman rank correlation between ASV and MTSI rankings was ρ = 0.763, indicating a strong positive association between the two rankings. Because lower values are desirable for both ASV and MTSI, this relationship indicates that genotypes performing favourably for grain-yield stability tended, in general, to occupy favourable positions under the multi-trait criterion as well. Of the ten genotypes ranked highest according to ASV, seven also occurred among the ten highest-ranked genotypes according to MTSI: IT17K-1095-2-2, IT17K-1403-1-1, IT17K-1802-1, IT17K-2024-4, IT17K-849-2-1, Padi-tuya, and SAC-20KTA-45. This substantial overlap suggests that grain-yield stability contributed importantly to the overall multi-trait ranking, while also demonstrating that the two approaches were not interchangeable.

The differences between the methods are particularly informative for breeding decisions. The five genotypes selected using the combined grain-yield and ASV criterion were Tuzievallenga, IT17K-2030-2, IT17K-1095-2-2, IT17K-849-2-1, and IT17K-1802-1, whereas the five lowest MTSI values were recorded for IT17K-1095-2-2, IT17K-849-2-1, IT17K-1403-1-1, IT17K-2024-4, and Tuzievallenga. Three genotypes, IT17K-1095-2-2, IT17K-849-2-1, and Tuzievallenga were common to both groups. In contrast, IT17K-2030–2 and IT17K-1802–1 were unique to the yield–stability group, whereas IT17K-1403-1–1 and IT17K-2024–4 were unique to the MTSI group. Thus, the two approaches converged on some genotypes but emphasized different breeding attributes in others.

IT17K-2030–2 provides the clearest example of this difference. It ranked first for ASV, indicating the lowest grain-yield interaction among the complete genotypes, but ranked ninth for MTSI. Its relatively high biomass yield (3382.2 kg ha^−1^) and hundred-seed weight (22.9 g) were favourable individual characteristics, but its overall MTSI position indicates that these advantages did not translate into the closest overall match to the multi-trait ideotype. This illustrates an important limitation of single-trait stability selection: a genotype can be exceptionally stable for grain yield while having a less favourable combination of other breeding traits. Recent cowpea research has similarly emphasized the value of integrating multiple traits when selecting genotypes under environmental stress, rather than relying on grain yield or stability alone (Ps et al., 2026).

Conversely, IT17K-1403-1–1 and IT17K-2024–4 illustrate the additional genotypes identified when selection was based on the broader ideotype. Their ASV ranks were third and fifth, respectively, and their MTSI ranks were also highly favourable, at third and fourth. These results indicate that relatively stable grain yield can coincide with a favourable overall combination of traits. IT17K-1095-2–2 showed the strongest convergence between the two approaches, ranking second for ASV and first for MTSI, while IT17K-849-2–1 ranked sixth for ASV and second for MTSI. Such convergence provides stronger evidence of their potential value than reliance on either criterion alone.

The position of Tuzievallenga is also noteworthy. It ranked fourth for ASV and fifth for MTSI while simultaneously recording the highest mean grain yield. Thus, unlike IT17K-2030-2, its high productivity was accompanied by favourable positions under both grain-yield stability and multi-trait selection. This convergence, together with its GGE performance across three environments, makes Tuzievallenga one of the most consistently supported genotypes in the present study. However, the agreement among statistical criteria should be interpreted as evidence supporting its performance within the environments evaluated rather than as independent validation of the genotype.

The relatively strong correlation between ASV and MTSI should therefore not be interpreted as evidence that one method is redundant. Rather, the results demonstrate that AMMI/ASV and MTSI answer related but different breeding questions. AMMI/ASV is useful when the primary objective is to identify genotypes that combine grain yield with low G×E interaction, whereas MTSI is more appropriate when breeders seek simultaneous improvement in several traits and wish to evaluate genotypes according to a defined ideotype. Olivoto et al. (2019) emphasized that multi-trait selection can improve the efficiency of genotype selection when several traits contribute to the breeding objective.

This complementary interpretation is consistent with recent cowpea research, where multi-trait stability indices have been used alongside AMMI and GGE approaches to identify genotypes combining productivity, stability and secondary agronomic characteristics. Such approaches are particularly relevant for cowpea because breeding objectives extend beyond grain yield to include maturity, seed characteristics, biomass and stress adaptation (Ps et al., 2026).

Overall, the present results indicate that no single selection criterion identified all of the most desirable genotypes. The overlap among IT17K-1095-2-2, IT17K-849-2–1 and Tuzievallenga provides particularly strong evidence of their favourable overall performance, whereas the differences between the two selection approaches reveal additional breeding materials with useful trait combinations. AMMI/ASV and MTSI should therefore be regarded as complementary selection tools: the former emphasizes grain-yield stability, while the latter broadens selection to simultaneous performance and stability across multiple agronomic traits. This integrated interpretation provides a stronger basis for deciding which genotypes should be advanced for on-farm evaluation and which may be retained as potential parents in subsequent breeding programmes.

### 4.8 Breeding implications and prospects for on-farm advancement

The combined results of AMMI, GGE and MTSI provide a useful basis for prioritizing genotypes for the next stage of evaluation and breeding. No single selection criterion identified all desirable genotypes, but several materials showed consistent evidence of favourable performance. Tuzievallenga was particularly notable for combining the highest mean grain yield with relatively favourable stability and a high MTSI ranking, while IT17K-1095-2–2 and IT17K-849-2–1 were consistently identified among the leading genotypes under both yield–stability and multi-trait selection. IT17K-1403-1–1 and IT17K-2024-4, although not among the top five under the combined yield–ASV criterion, ranked highly under MTSI, indicating desirable combinations of traits beyond grain yield stability. These genotypes therefore represent contrasting but complementary candidates for advancement. Recent drought-response studies have likewise identified IT17K-849-2–1 and IT17K-1095-2–2 as promising genetic resources under water-limited conditions, supporting their continued evaluation and potential use as parents in breeding programmes targeting drought resilience (Tengey et al., 2026).

The appropriate next step for the most promising materials is on-farm testing under farmer-managed conditions, rather than relying solely on researcher-managed trials. On-farm evaluation provides an opportunity to determine whether the performance observed under experimental conditions is maintained under the range of management practices and production conditions encountered by farmers. It also allows agronomic performance to be considered together with farmer acceptance and other characteristics that may not be adequately captured by statistical selection indices. Evidence from northern Ghana demonstrates the value of participatory varietal selection in cowpea improvement, with farmers evaluating traits including grain yield, maturity, pod characteristics, grain colour and size, and biomass yield (Jinbaani et al., 2023).

Farmer participation is particularly relevant because genotype preference is not determined by grain yield alone. Research involving cowpea farmers in northern Ghana found that drought tolerance, short cooking time and pest resistance were highly preferred traits among both women and men, while preferences for characteristics such as seed colour and earliness could differ by gender (Jinbaani et al., 2023). Earlier participatory work in Ghana also demonstrated that farmers could effectively contribute to selection of cowpea materials based on traits such as seed size, pod characteristics and maturity (Egbadzor et al., 2015). Consequently, on-farm evaluation of the promising genotypes identified in the present study should incorporate farmer assessment alongside measurements of grain yield, maturity, seed characteristics, biomass and adaptation (Jinbaani et al., 2023).

The breeding value of the materials should also be considered independently of their ranking for direct advancement. Genotypes that do not occupy the highest overall position may possess specific traits that are useful for hybridization. For example, IT17K-2030–2 combined very low ASV with high biomass yield and relatively large seed size, whereas other genotypes contributed high grain yield, favourable maturity or desirable multi-trait profiles. Such complementary attributes could be exploited through crossing to develop progenies combining yield, stability, earliness, seed size and biomass production. This approach is consistent with participatory and trait-based cowpea breeding in Ghana, where breeding objectives have increasingly incorporated farmer-identified traits alongside agronomic performance.

The distinction between genotypes for advancement and genotypes for use as parents is therefore important. Selection for on-farm testing should prioritize genotypes with consistent evidence of favourable performance across the analytical approaches, whereas parental selection can deliberately retain materials with complementary strengths even when their overall ranking is lower. This is particularly relevant for maintaining genetic diversity within the breeding programme and for combining attributes that are unlikely to occur at desirable levels in a single genotype.

Overall, the findings support a two-pronged breeding strategy. Genotypes with convergent evidence of high yield, stability and favourable multi-trait performance particularly Tuzievallenga, IT17K-1095-2–2 and IT17K-849-2–1 should be prioritized for on-farm evaluation to determine their performance, adaptation and acceptance under farmer-managed conditions. Other genotypes with valuable individual attributes should be retained strategically as potential parents for hybridization. Such an approach recognizes that cultivar development requires both reliable selection of superior candidates and continued generation of new genetic combinations to address the diverse production environments and farmer preferences encountered in cowpea production.

## 5 Conclusions and recommendations

The study demonstrated significant effects of environment, genotype and genotype × environment interaction (G×E) on grain yield of cowpea genotypes evaluated across five location-year environments in Ghana, highlighting the importance of environmental variation in genotype selection. AMMI analysis showed that IPCA1 and IPCA2 accounted for 68.51% and 23.64% of the G×E interaction, respectively, explaining 92.15% cumulatively. Tuzievallenga recorded the highest mean grain yield (1149.15 kg ha^−1^) and relatively favourable stability, while IT17K-2030–2 had the lowest ASV (114.54). Five genotypes, Tuzievallenga, IT17K-2030-2, IT17K-1095-2-2, IT17K-849-2–1 and IT17K-1802–1 were identified as high-yielding and stable. GGE analysis revealed one common sector comprising Damongo_2024, Manga_2025 and Nyankpala_2024, with Tuzievallenga as the winning genotype, while Wa_2025 and Nyankpala_2025 showed distinct genotype responses. MTSI provided complementary evidence, with IT17K-1095-2-2, IT17K-849-2-1, IT17K-1403-1-1, IT17K-2024–4 and Tuzievallenga having the lowest index values. The convergence of selection criteria particularly identified Tuzievallenga, IT17K-1095-2–2 and IT17K-849-2–1 as promising materials. These genotypes should be prioritized for farmer-managed on-farm testing to assess their adaptation, performance and acceptance under practical production conditions before varietal advancement, while genotypes with complementary desirable traits, including IT17K-2030-2, should be considered as parents in hybridization programmes. The GGE sectors identified should be regarded as preliminary adaptation patterns rather than definitive mega-environments.

## Data Availability

The original contributions presented in the study are included in the article/**Supplementary Material**. Further inquiries can be directed to the corresponding author.
