# Supplementary material for: Integrated AMMI, GGE Biplot, and MTSI analyses reveal yield stability and adaptation patterns in cowpea in Ghana
Source: Front Plant Sci. 2026 Oct 9;17:1897494. doi: 10.3389/fpls.2026.1897494 (PMC13690774; doi:10.3389/fpls.2026.1897494)
Supplement: Supplementary file 1 [file Table1.docx]

**Table S1a. Monthly and seasonal precipitation during the 2024 and 2025 cropping seasons compared with 30-year averages**

| **Location** | **Year** | **July** | **August** | **September** | **October** | **Total** |
| --- | --- | --- | --- | --- | --- | --- |
| **Nyankpala** | 2024 | 115.99 | 146.87 | 189.34 | 168.73 | **620.93** |
|  | 2025 | 109.93 | 119.15 | 250.54 | 111.25 | **590.87** |
|  | 1994–2023 average | 169.79 | 183.62 | 207.75 | 112.06 | **673.22** |
|  | Departure, 2024 (%) | −31.7 | −20.0 | −8.9 | +50.6 | **−7.8** |
|  | Departure, 2025 (%) | −35.3 | −35.1 | +20.6 | −0.7 | **−12.2** |
| **Damongo** | 2024 | 63.47 | 149.50 | 164.11 | 193.22 | **570.30** |
|  | 2025 | 82.78 | 72.83 | 241.86 | 110.64 | **508.11** |
|  | 1994–2023 average | 132.81 | 159.50 | 185.65 | 111.92 | **589.88** |
|  | Departure, 2024 (%) | −52.2 | −6.3 | −11.6 | +72.6 | **−3.3** |
|  | Departure, 2025 (%) | −37.7 | −54.3 | +30.3 | −1.1 | **−13.9** |
| **Manga** | 2024 | 195.37 | 220.07 | 162.92 | 84.42 | **662.78** |
|  | 2025 | 175.84 | 247.32 | 176.92 | 58.16 | **658.24** |
|  | 1994–2023 average | 206.30 | 260.27 | 165.51 | 73.06 | **705.14** |
|  | Departure, 2024 (%) | −5.3 | −15.4 | −1.6 | +15.5 | **−6.0** |
|  | Departure, 2025 (%) | −14.8 | −5.0 | +6.9 | −20.4 | **−6.7** |
| **Wa** | 2024 | 110.78 | 218.78 | 146.98 | 110.86 | **587.40** |
|  | 2025 | 173.22 | 119.67 | 227.13 | 53.97 | **573.99** |
|  | 1994–2023 average | 153.46 | 198.69 | 196.14 | 84.68 | **632.97** |
|  | Departure, 2024 (%) | −27.8 | +10.1 | −25.1 | +30.9 | **−7.2** |
|  | Departure, 2025 (%) | +12.9 | −39.8 | +15.8 | −36.3 | **−9.3** |

**Note:** Long-term monthly averages are based on 1994–2023 data. Departure in precipitation (%) = [(observed precipitation − 30-year average precipitation) / 30-year average precipitation] × 100. Negative values indicate precipitation deficits relative to the long-term average; positive values indicate above-average precipitation.

**Table S1b. Monthly and seasonal mean temperature during the 2024 and 2025 cropping seasons compared with 30-year averages**

| **Location** | **Year** | **July** | **August** | **September** | **October** | **Mean** |
| --- | --- | --- | --- | --- | --- | --- |
| **Nyankpala** | 2024 | 26.62 | 26.61 | 26.24 | 26.87 | **26.59** |
|  | 2025 | 26.35 | 25.62 | 26.01 | 27.51 | **26.37** |
|  | 1994–2023 average | 26.17 | 25.49 | 25.78 | 26.99 | **26.11** |
|  | Departure, 2024 (°C) | +0.45 | +1.12 | +0.46 | −0.12 | **+0.48** |
|  | Departure, 2025 (°C) | +0.18 | +0.13 | +0.23 | +0.52 | **+0.26** |
| **Damongo** | 2024 | 26.59 | 26.46 | 26.02 | 26.97 | **26.51** |
|  | 2025 | 26.19 | 25.75 | 26.00 | 27.78 | **26.43** |
|  | 1994–2023 average | 25.86 | 25.31 | 25.59 | 26.89 | **25.91** |
|  | Departure, 2024 (°C) | +0.73 | +1.15 | +0.43 | +0.08 | **+0.60** |
|  | Departure, 2025 (°C) | +0.33 | +0.44 | +0.41 | +0.88 | **+0.52** |
| **Manga** | 2024 | 27.03 | 26.14 | 26.31 | 27.58 | **26.77** |
|  | 2025 | 26.64 | 25.52 | 26.08 | 28.59 | **26.71** |
|  | 1994–2023 average | 26.97 | 25.60 | 26.11 | 28.09 | **26.69** |
|  | Departure, 2024 (°C) | +0.06 | +0.54 | +0.20 | −0.51 | **+0.07** |
|  | Departure, 2025 (°C) | −0.33 | −0.08 | −0.03 | +0.50 | **+0.02** |
| **Wa** | 2024 | 26.07 | 25.49 | 25.36 | 26.23 | **25.79** |
|  | 2025 | 25.22 | 24.83 | 25.14 | 26.65 | **25.46** |
|  | 1994–2023 average | 25.50 | 24.71 | 25.09 | 26.38 | **25.42** |
|  | Departure, 2024 (°C) | +0.57 | +0.79 | +0.27 | −0.15 | **+0.37** |
|  | Departure, 2025 (°C) | −0.28 | +0.12 | +0.04 | +0.27 | **+0.04** |

**Note:** Long-term monthly averages are based on 1994–2023 data. Departure in temperature (°C) = observed temperature − 30-year average temperature. Positive values indicate warmer conditions and negative values indicate cooler conditions relative to the long-term average.

**Table S2. Classification of cowpea genotypes according to mean grain yield and AMMI Stability Value (ASV)**

| **Yield–stability category** | **Genotypes** | **Interpretation** |
| --- | --- | --- |
| **High yield / Highly stable** | **Tuzievallenga; IT17K-2030-2; IT17K-1095-2-2** | Genotypes combining relatively high mean grain yield with low ASV, indicating comparatively consistent performance across environments. **Tuzievallenga** had the highest mean grain yield (1149.1 kg ha⁻¹) with good stability (ASV = 367.96), whereas **IT17K-2030-2** had the lowest ASV (114.54), indicating the greatest stability among the evaluated genotypes. |
| **High yield / Moderately stable** | **IT17K-849-2-1; IT17K-1802-1; IT17K-1809-4** | Genotypes with relatively high mean grain yield and intermediate ASV values. Their performance indicates good yield potential but greater genotype × environment interaction than the highly stable group. **IT17K-849-2-1** yielded 954.2 kg ha⁻¹ (ASV = 449.24), while **IT17K-1802-1** yielded 1081.2 kg ha⁻¹ (ASV = 594.18). |
| **High yield / Less stable** | **SARI-tuya; IT17K-1266-2-1; KVX782-1; IT17K-1367-2-3** | Genotypes with relatively high yield potential but comparatively large ASV values, indicating greater variation in performance across environments. **SARI-tuya** yielded 1093.2 kg ha⁻¹ (ASV = 1759.89), while **IT17K-1266-2-1** had the highest ASV (2261.51), indicating substantial genotype × environment interaction. |
| **Low yield / Highly stable** | **IT17K-1403-1-1; IT17K-2024-4** | Genotypes with relatively low mean grain yield but low ASV values, indicating consistent performance across environments. **IT17K-1403-1-1** yielded 692.2 kg ha⁻¹ (ASV = 329.45), whereas **IT17K-2024-4** yielded 608.9 kg ha⁻¹ (ASV = 435.96). |
| **Low yield / Moderately stable** | **SAC-20KTA-45; IT17K-1279-3-1; Padi-tuya; IT17K-1016-1-1; IT99K-573-1-1; Wangkae** | Genotypes characterized by relatively low mean grain yield and intermediate ASV values. Their combination of yield and stability provides limited evidence for selection as broadly adapted high-yielding genotypes, although individual lines may remain useful as breeding material for specific traits. |
| **Low yield / Less stable** | **IT17K-1707-2-2** | Genotype characterized by relatively low mean grain yield and high ASV, indicating both limited yield performance and substantial variation across environments. Mean grain yield was 700.8 kg ha⁻¹ and ASV was 2067.22. |

**Note:** Yield and stability categories should be interpreted according to the thresholds defined in the Materials and Methods. Lower ASV values indicate greater stability, whereas higher ASV values indicate greater contribution to genotype × environment interaction. The classification concerns mean performance and stability across the test environments and does not by itself establish adaptation to specific production zones.

**Table S3. Top-performing cowpea genotypes based on mean grain yield and AMMI Stability Value (ASV)**

| **Rank** | **Genotype** | **Mean grain yield (kg ha⁻¹)** | **ASV** | **Yield category** | **Stability category** | **Interpretation** |
| --- | --- | --- | --- | --- | --- | --- |
| **1** | **Tuzievallenga** | **1149.1** | **367.96** | High | Highly stable | **Best overall performer based on the combination of mean grain yield and stability.** It recorded the highest mean grain yield while maintaining a relatively low ASV, indicating strong yield potential combined with comparatively consistent performance across environments. |
| **2** | **IT17K-2030-2** | **791.2** | **114.54** | High | Highly stable | **Most stable genotype based on ASV.** Its very low ASV indicates highly consistent performance across environments, although its mean grain yield was lower than that of the highest-yielding genotypes. |
| **3** | **IT17K-1095-2-2** | **735.8** | **232.55** | High | Highly stable | **Stable performer with moderate grain yield.** The low ASV indicates consistent performance across environments, making this genotype particularly relevant where yield consistency is an important selection criterion. |
| **4** | **IT17K-849-2-1** | **954.2** | **449.24** | High | Moderately stable | **High-yielding genotype with moderate stability.** Its relatively high grain yield combined with an intermediate ASV indicates good yield potential accompanied by greater environmental responsiveness than the highly stable genotypes. |
| **5** | **IT17K-1802-1** | **1081.2** | **594.18** | High | Moderately stable | **Very high-yielding genotype with moderate stability.** It ranked second for mean grain yield but had a higher ASV than the most stable genotypes, indicating greater genotype × environment interaction. |

**Note:** Rankings are based on the combined consideration of mean grain yield and ASV. ASV measures the magnitude of genotype × environment interaction. Above average grain yield (> 735.8 kg/ha) value is considered high while below average grain yield (< 735.8 kg/ha) is considered low.

**Table S4. Comparison of the top five genotypes ranked by grain yield and Multi-Trait Stability Index (MTSI)**

| **Selection criterion** | **Rank 1** | **Rank 2** | **Rank 3** | **Rank 4** | **Rank 5** |
| --- | --- | --- | --- | --- | --- |
| **Grain yield only** | **Tuzievallenga** (1149.1 kg ha⁻¹) | **SARI-tuya** (1093.2 kg ha⁻¹) | **IT17K-1802-1** (1081.2 kg ha⁻¹) | **IT17K-849-2-1** (954.2 kg ha⁻¹) | **IT17K-1266-2-1** (829.4 kg ha⁻¹) |
| **MTSI** | **IT17K-1095-2-2** (2.035) | **IT17K-849-2-1** (2.148) | **IT17K-1403-1-1** (2.409) | **IT17K-2024-4** (2.723) | **Tuzievallenga** (2.844) |

**Note:** Grain-yield ranking is based solely on mean grain yield, whereas MTSI ranking reflects simultaneous consideration of the traits included in the multi-trait selection analysis. MTSI values should be interpreted according to the direction and scaling specified for the particular MTSI analysis. The difference between the two rankings illustrates that genotype selection based on multiple traits can differ substantially from selection based on grain yield alone.

**Table S5. Top-ranked genotypes under the Multi-Trait Stability Index (MTSI) selection criterion**

| **Rank** | **Genotype** | **MTSI score** | **Key strengths** | **Main limitations** | **Interpretation** |
| --- | --- | --- | --- | --- | --- |
| **1** | **IT17K-1095-2-2** | **2.035** | Excellent stability (ASV = 232.55); balanced multi-trait performance; consistent grain yield (735.8 kg ha⁻¹); good harvest index (0.172); moderate maturity (70.7 days) | Moderate grain yield relative to the highest-yielding genotypes; moderate seed weight (17.9 g) | **Highest-ranked genotype under the reported MTSI analysis.** Its principal strength is the balance among yield, stability and other agronomic traits rather than maximum grain yield alone. |
| **2** | **IT17K-849-2-1** | **2.148** | High grain yield (954.2 kg ha⁻¹); high dry biomass (3345.6 kg ha⁻¹); good harvest index (0.183); early maturity (69.5 days); good stability (ASV = 449.24) | Relatively small seed weight (16.4 g) | **Strong multi-trait performer combining high yield, relatively good stability, biomass production and early maturity.** |
| **3** | **IT17K-1403-1-1** | **2.409** | Excellent stability (ASV = 329.45); high dry biomass (3139.7 kg ha⁻¹); good seed weight (18.4 g); consistent performance across environments | Moderate grain yield (692.2 kg ha⁻¹); low harvest index (0.148); relatively late maturity (71.6 days) | **Useful multi-trait source of stability and biomass.** Its lower grain yield limits its value when grain productivity is the primary objective. |
| **4** | **IT17K-2024-4** | **2.723** | Good stability (ASV = 435.96); stable yield performance; moderate harvest index (0.152) | Low grain yield (608.9 kg ha⁻¹); low dry biomass (2548.3 kg ha⁻¹); small seed weight (17.1 g); relatively late maturity (71.7 days) | **Potentially useful as a stable breeding source**, particularly where consistency is prioritized, although its lower yield limits its value as a direct high-yielding candidate. |

**Table S6. Rank comparison of cowpea genotypes based on AMMI Stability Value (ASV) and Multi-Trait Stability Index (MTSI)**

| **Genotype** | **ASV Rank** | **MTSI Rank** |
| --- | --- | --- |
| IT17K-1016-1-1 | 11 | 17 |
| IT17K-1095-2-2 | 2 | 1 |
| IT17K-1266-2-1 | 19 | 12 |
| IT17K-1279-3-1 | 9 | 13 |
| IT17K-1367-2-3 | 16 | 20 |
| IT17K-1403-1-1 | 3 | 3 |
| IT17K-1707-2-2 | 17 | 16 |
| IT17K-1802-1 | 7 | 6 |
| IT17K-1809-4 | 12 | 15 |
| IT17K-2024-4 | 5 | 4 |
| IT17K-2030-2 | 1 | 9 |
| IT17K-849-2-1 | 6 | 2 |
| IT99K-573-1-1 | 13 | 11 |
| Kirkhouse Benga | — | 18 |
| KVX782-1 | 18 | 19 |
| Padi-tuya | 10 | 7 |
| SAC-20KTA-45 | 8 | 10 |
| SARI-tuya | 15 | 8 |
| Tuzievallenga | 4 | 5 |
| Wangkae | 14 | 14 |

**Note:** ASV ranks are based on the 19 genotypes with complete observations across all five environments; Kirkhouse Benga was excluded from the AMMI/ASV analysis because its Manga_2025 observation was missing. MTSI ranks are based on all 20 genotypes.
